# Structure prediction of linear and cyclic peptides using CABS-flex

**DOI:** 10.1093/bib/bbae003

**Published:** 2024-02-01

**Authors:** Aleksandra Badaczewska-Dawid, Karol Wróblewski, Mateusz Kurcinski, Sebastian Kmiecik

**Affiliations:** Genome Informatics Facility, Office of Biotechnology, Iowa State University, Ames, 50011 IA, USA; Biological and Chemical Research Center, Faculty of Chemistry, University of Warsaw, Pasteura 1, 02-093 Warsaw, Poland; Biological and Chemical Research Center, Faculty of Chemistry, University of Warsaw, Pasteura 1, 02-093 Warsaw, Poland; Biological and Chemical Research Center, Faculty of Chemistry, University of Warsaw, Pasteura 1, 02-093 Warsaw, Poland

**Keywords:** peptide, cyclic peptides, drug design, structural modeling, multiscale modeling

## Abstract

The structural modeling of peptides can be a useful aid in the discovery of new drugs and a deeper understanding of the molecular mechanisms of life. Here we present a novel multiscale protocol for the structure prediction of linear and cyclic peptides. The protocol combines two main stages: coarse-grained simulations using the CABS-flex standalone package and an all-atom reconstruction-optimization process using the Modeller program. We evaluated the protocol on a set of linear peptides and two sets of cyclic peptides, with cyclization through the backbone and disulfide bonds. A comparison with other state-of-the-art tools (APPTEST, PEP-FOLD, ESMFold and AlphaFold implementation in ColabFold) shows that for most cases, AlphaFold offers the highest resolution. However, CABS-flex is competitive, particularly when it comes to short linear peptides. As demonstrated, the protocol performance can be further improved by combination with the residue–residue contact prediction method or more efficient scoring. The protocol is included in the CABS-flex standalone package along with online documentation to aid users in predicting the structure of peptides and mini-proteins.

## INTRODUCTION

In recent years, we have seen a renewed interest in using peptides as possible drug candidates [1]. In the process of designing peptide drugs, structural knowledge of peptide or protein–peptide interactions is invaluable [2]. Recently, we have seen a breakthrough in the field of protein structure prediction. The AlphaFold2 method has been developed that can predict protein structures with accuracy similar to the experimental methods [3]. Methods, such as AlphaFold2, are trained on static protein models from experimental databases. Unfortunately, experimental knowledge of the structural repertoire of peptide or peptide–protein interactions is much smaller than that of intra-protein or protein–protein interactions. The problem of predicting the structure of bound peptides can be solved by treating the peptide interactions exactly as a protein monomer fragment [4]. However, the transient and dynamic nature of the peptide interactions may not be well captured here (in contrast to usually well-defined intra-protein or protein–protein interactions).

Peptide cyclization, either through the backbone or disulfide bonds, offers several advantages in peptide drug design like increased stability, bioavailability or improved receptor binding [5–7]. Along with experimental methods, the computational prediction of linear and cyclic peptide structures can be incorporated into the drug discovery process [8].

In the last decade, we introduced the CABS-flex method for fast modeling of protein structure flexibility [9–11]. The tool has gone through several development cycles and is currently available as web server 2.0 version [12], as well as the CABS-flex standalone application that provides command-line access and control over the simulation process [13]. This paper introduces an innovative protocol for *de novo* peptide structure prediction using the CABS-flex standalone application. The protocol allows modeling peptide structure starting solely from amino acid sequence. The optimized simulation settings enable accurate structure prediction for both linear and cyclic peptides, with backbone- or disulfide- cyclization introduced through distance constraints. Similarly, any other knowledge of amino acid residue–residue contacts can be introduced into the modeling process. The presented protocol is intended for easy use, even with limited computational resources, and can be incorporated into various multiscale pipelines.

## METHODS

### Benchmark datasets

In this study, we used a comprehensive collection of 159 peptides, including 97 linear and 62 cyclic ones. It represents a non-redundant superset compiled from benchmarks known in the literature [14, 15] that has been further augmented with a unique set of peptides closed by a backbone [16, 17]. The array of sequences covers a wide range of peptide sizes (from 9 to 49 amino acids) and shapes (linear, cyclic). They also differ by their propensity to form a secondary structure (helices, β-sheets) and the cyclization driving force (backbone covalent bond, disulfide bridge). Thus, in our view, such a heterogeneous dataset provides a reliable benchmark for testing peptide structure prediction protocol. The reference state, used solely for assessing the modeling efficiency, was selected as a PDB model with the lowest RMSD calculated relative to the top-scored model from the simulation trajectory (more information in Supplementary Materials).

### Linear peptides spanning various lengths

Linear peptides were classified into three subsets based on the length of their amino acid (AA) sequence: (42) short up to 20 AA, (26) medium in range of 27–40 AA and (29) long up to 50 AA. The peptides were taken from the recent work of the APPTEST [14] and complemented with a set of longer peptides (above 40 AA) originally introduced as a benchmark by the PEP-FOLD team [15]. The known experimental structures of these peptides differ significantly in their propensity to form a well-folded structure (20–80% of secondary structure, favoring either mainly helices or mainly β-sheets, the detailed information is provided in Tables S3, S4 and S5 of the Supplementary Materials).

### Disulfide and backbone cyclic peptides

Cyclic peptides were classified into two subsets based on the cyclization type: disulfide- and backbone-cyclized peptides. The disulfide-cyclized set contains 34 rather short peptides (between 10 and 30 amino acids that are mostly unstructured or form sparse helices), introduced by the authors of the PEP-FOLD 2.0 [18]. The backbone-cyclized set contains 28 peptide examples, mostly cyclotides [19], derived from the Cybase [16, 17] for the purpose of this work. The secondary structure content is low, averaging 21%. More details are provided in Tables S6 (disulfide set) and S7 (backbone set) of the Supplementary Materials.

### CABS-flex standalone application

Our approach uses CABS, a medium resolution coarse-grained (CG) protein model with an efficient sampling in the Monte Carlo Dynamics scheme. CABS model has demonstrated broad applications in protein modeling, simulation of dynamics and disordered states [20–22] and protein–peptide docking [23–25]. In this work, we employed CABS-flex standalone application from the broad family of well-established CABS-based tools. It is a Python package for fast simulations of protein structure flexibility [13]. The repository of the CABS-flex package is available at https://bitbucket.org/lcbio/cabsflex/. A novel option has been introduced as a part of this study to enable fast *de novo* modeling of peptides up to 100 amino acids in length.

### Using CABS-flex for peptide modeling

**Figure 1:**
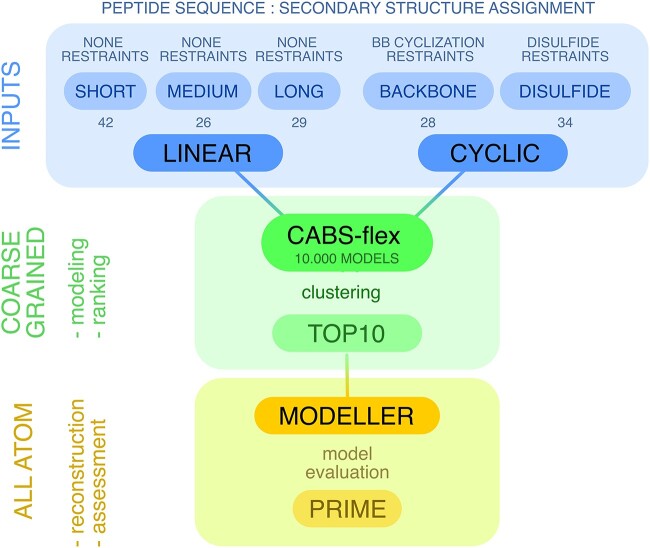
The protocol for peptide structure prediction and the benchmark characterization: (i) input preparation and reference states (top row in blue), (ii) coarse-grained (CG) modeling using CABS-flex (middle row in green) and (iii) reconstruction to all-atom (AA) representation using Modeller-based approach and assessment of final high-resolution results (bottom row in yellow). The benchmark includes 159 peptides divided into two major groups: linear (97) and cyclic (62). For each case, the CABS-flex generates 10 000 models in alpha-carbon representation only. Representative 10 (top10) models are selected using structural clustering. Next, the set of top10 is reconstructed to all-atom resolution and optimized using a Modeller-based approach. In CABS-flex, *prime* and *best* models are the highest accuracy models out of the top10 and 10 000 models, respectively.

The proposed protocol uses the CABS-flex standalone application (see Figure 1) with the following inputs:

Amino acid sequence is the only explicitly required input for *de novo* modeling of peptide structure using CABS-flex. In this work, the sequence information was extracted from the ‘SEQRES’ records of the PDB files deposited in the Protein Data Bank to avoid missing residues (not experimentally determined). In the primary sequence, the non-standard amino acids were replaced with standard equivalents, as required by the CABS-flex application.
Secondary structure assignment in 3-letter notation (C - coil, H - helix, E - extended). In this work, we assigned secondary structure from experimental PDB structures using the DSSP [26] algorithm (see Tables S3–S7 of the Supplementary Materials). The secondary structure input is incorporated in the knowledge-based force field of the CABS model as subtle preferences of the local chain geometry and residue–residue contacts (see details in [27]).
Information on internal disulfide bonds favoring a specified pair of cysteine residues to stay close in space. Used only in peptides from cyclic datasets and introduced as side-chain distance restraints, for the syntax description see Table S1 in Supplementary Materials. The information of internal disulfide bonds was identified from ‘SSBOND’ records in the header of the PDB files.
Information on cyclization through the backbone ends. Used only in peptides from backbone cyclized dataset and introduced as alpha-carbon distance restraints, for the syntax description see Table S1 in Supplementary Materials.
Information on residue–residue contact predictions. In a separate series of simulations, we introduced extra distance restraints using state-of-the-art sequence-based contact predictors. We employed RaptorX Contact [28], to identify regions essential for maintaining protein structure [29]. Unfortunately, this method is also limited by a lower bound of the sequence length. Thus, we could only generate results for peptides longer than 20 AA that cover most of the medium and long linear and cyclic via backbone datasets (in total, 81 of 159 peptides). The predicted contacts were used as an input in the CABS-flex in the form of alpha-carbon distance restraints (see the syntax in Table S1). A list of predicted contacts, together with contacts calculated from experimental structures, is provided in Tables S3–S7 of the Supplementary Materials.

Below we present an example syntax for the CABS-flex standalone command. Input settings for each of the simulations are shown in Table S1, whereas the rest of commands for complete series of simulations are given in Table S2 of Supplementary Materials.

CABSflex -i *AA_SEQUENCE*:*SECONDARY_STRUCTURE* -R ../reference.pdb:*CHAIN.*

-a 20 -y 50 -D 0.1 -t 3.0 1.0 -r 10 -f 0 -N -z 123,456.

-o RFCM -M -S -C.

—sc-rest-file *disulfide.txt* —ca-rest-file *contacts.txt.*

The CABS-flex documentation provides broad guidance through the options along with explanatory examples: https://bitbucket.org/lcbio/cabsflex/wiki/Home#markdown-header-2-cabsflex-options

The CABS-flex simulation returns 10 000 models in C-alpha representation by default (as a joint batch of 10 replicas generating 1000 conformations each). In an automated procedure available within CABS-flex standalone, 1000 models top-scored by CABS energy are structurally clustered resulting in the 10 most dense clusters. The representative model (medoid) of each group creates the top10 subset.

### Using Modeller for all-atom reconstruction and optimization

The structure reconstruction step in the presented protocol (see Figure 1) involved a Modeller-based approach, commuting from the CA-trace to all-atom representation. Modeller is a comparative protein structure modeling program that utilizes spatial restraints to determine the positions of non-hydrogen atoms [30]. It is a command-line utility that can be seamlessly integrated within multiscale pipelines. Consequently, CABS-based tools offer a built-in feature for automatic all-atom reconstruction using this method. In this study, we employed an enhanced version of our in-house Modeller-based protocol (*ca2all* python script). The protocol uses coarse-grained models as templates and automodel class from Modeller to generate a comprehensive set of restraints to reconstruct all heavy atoms and effectively address unphysical distortions in coarse-grained models, such as Ca-traces that came out of CABS-flex. The reconstruction protocol was described previously [31, 32] and the source code is available at https://bitbucket.org/lcbio/ca2all.

Below, we present an example syntax for the *ca2all* utilization in the command line. Note that we used the same CA-model obtained from the coarse-grained modeling step as both the input (−i option) and template (−t option). Optionally, the –k flag (or —add-hydrogens) includes hydrogen atoms in the final all-atom structure (returned with –o option).

python ca2all.py -i *model_CA.pdb* -t *model_CA.pdb* -o *model_AA.pdb.*

## RESULTS AND DISCUSSION

To thoroughly assess CABS-flex's predictive accuracy, we conducted a comparative analysis against four alternative methods: APPTEST [14], PEP-FOLD [15], ESMFold [33] and AlphaFold [3]. The AlphaFold was used in the form of a ColabFold notebook, an easy-to-use environment for fast and convenient structure predictions [34]. What is important, ColabFold has been adapted for modeling peptides shorter than 16 AA, as opposed to the standalone AlphaFold version.

For each peptide, we describe and compare the two models—*prime* and *best*. In the case of CABS-flex, *prime* is the highest accuracy model out of 10 top-scored models (see Figure 1). *Prime* models for APPTEST were chosen out of top 10 and for PEP-FOLD out of top five (see details in [14, 15]). AlphaFold was configured to generate five models and ESMFold produces only one model by default. The *best* model is the highest accuracy model out of all models created by each method. It serves as a theoretical measure of how good a method is in conformational sampling. Comparing prime to best illustrates how effectively a method can rank and select the most accurate models from a vast pool of structures.

### Structure prediction of linear peptides

In Table 1 and Table 2, we present the full comparison between CABS-flex, CABS-flex with RaptorX contacts, APPTEST and PEP-FOLD, AlphaFold and ESMFold. Tables S8, S9 and S10 of Supplementary Materials contain the full results for every linear peptide in the benchmark.

**Table 1:** Comparison between CABS-flex, CABS-flex with RaptorX contacts (RX), APPTEST, PEP-FOLD, AlphaFold and ESMFold performances. The first three columns contain the name, the number of peptides and the range of peptide length for each of the datasets. The remaining 10 columns (tools) show the average modeling performance for prime and best models over the different datasets (in rows) measured as the Full Structure B-RMSD (backbone-RMSD) and Rigid Core^*^ B-RMSD (in the parenthesis) calculated on the all-atom results of modeling. The last row shows the average modeling performance over all the peptides. In case of CABS-flex with RaptorX, if there were no additional contacts predicted, the scores were copied from the CABS-flex column in order to calculate the average

			CABS FLEX	CABS FLEX + RX	APP TEST	PEP FOLD	ALPHA FOLD	ESM FOLD	CABS FLEX	CABS FLEX + RX	APP TEST	PEP FOLD
dataset	N	L	PRIME						BEST			
			LINEAR									
SHORT	42	9–20	2.09 (1.48)	—	2.60 (1.70)	2.81 (2.00)	2.51 (1.72)	2.71 (1.88)	1.17 (0.70)	—	1.95 (1.36)	2.05 (1.64)
MEDIUM	26	27–40	5.44 (3.96)	4.81 (3.41)	4.49 (3.29)	5.91 (4.24)	2.80 (1.63)	3.59 (2.16)	3.51 (2.37)	3.05 (2.00)	3.19 (2.34)	3.63 (2.49)
LONG	29	41–49	5.36 (4.14)	4.59 (3.48)	—	5.07 (3.96)	3.62 (2.77)	4.53 (3.38)	4.00 (3.02)	3.56 (2.66)	—	3.75 (2.87)
			CYCLIC									
DISULFIDE	34	10–30	3.04 (2.92)	—	2.67 (2.43)	4.17 (4.24)	1.96 (2.02)	2.41 (2.17)	1.88 (1.80)	—	1.99 (1.86)	2.70 (2.66)
BACKBONE^*^^*^	28	14–39	4.83 (4.74)	4.06 (3.96)	1.57 (1.51)	5.78 (5.62)	2.14 (1.97)	3.63 (3.18)	3.27 (3.18)	2.82 (2.73)	1.49 (1.43)	4.19 (4.03)
			AVERAGE									
ALL	159	9–49	3.92 (3.54)	3.54 (3.10)	2.78 (2.19)	4.55 (4.04)	2.58 (2.04)	3.28 (2.62)	2.59 (2.29)	2.36 (2.03)	2.11 (1.73)	3.13 (2.78)

^*^Rigid core is defined as a peptide fragment that is not flexible (i.e. with residues displaying cRMSf values <1.5 Å in NMR ensembles or calculated fluctuations, see definition in [35]). ^*^^*^Some peptides from the BACKBONE dataset were used to train the APPTEST neural network. Results for each peptide from the BACKBONE dataset (including the information on using in the training set) are available in Table S12 of Supplementary Materials.

**Table 2:** Comparison between CABS-flex, CABS-flex with RaptorX contacts (RX), APPTEST, PEP-FOLD, AlphaFold and ESMFold performances for prime and best models in the different datasets. The results indicate the number of cases in which a particular method produces the highest quality result (lowest Full Structure B-RMSD) compared with other methods

		CABS FLEX	CABS FLEX + RX	APP TEST	PEP FOLD	ALPHA FOLD	ESM FOLD	CABS FLEX	CABS FLEX + RX	APP TEST	PEP FOLD
dataset	N	PRIME						BEST			
		LINEAR									
SHORT	42	13	—	4	4	10	11	35	—	5	2
MEDIUM	26	0	1	3	2	17	3	4	6	11	5
LONG	29	3	4	—	3	18	1	3	15	—	11
		CYCLIC									
DISULFIDE	34	5	—	4	2	18	5	22	—	10	2
BACKBONE^*^	28	1	0	14	0	13	1	1	0	27	0

^a^Some peptides from the BACKBONE dataset were used to train the APPTEST neural network (more information is presented in the Table S12 of Supplementary Materials).

For short-length peptides (between 9 and 20 AA) CABS-flex standalone outperforms the other methods in 13 cases out of 42 (AlphaFold—10, ESMFold—11, APPTEST—4, PEP-FOLD—4) in prime models; and 35 out of 42 cases (APPTEST—5, PEP-FOLD—2) in best models category (see Table 2). Figure 3 illustrates a few examples where the difference in prime models favored CABS-flex over AlphaFold. The upper row shows examples of short linear peptides (1ID6, 1L3Q) in which CABS-flex and experimental models do not contain regular secondary structure elements, in contrast to those predicted by AlphaFold. In the case of 1E0Q, the beta-sheet predicted by AlphaFold is only slightly tilted, whereas in the CABS-flex and the experimental structure, it is much more twisted. A possible reason for this may be AlphaFold's training dataset, which primarily consists of large proteins resolved through X-ray crystallography [3]. Consequently, in AlphaFold, peptides are often approached as compact protein fragments in a densely packed environment rather than unbound flexible entities with potentially distinct structures. Therefore, the simplicity of the peptide structure, combined with the computational efficiency of CABS-flex, makes it competitive with AlphaFold in predicting the structures of shorter flexible peptides, where the absence of extensive long-range interactions and global structural complexities diminishes the advantages of deep learning-based approaches.

**Figure 2:**
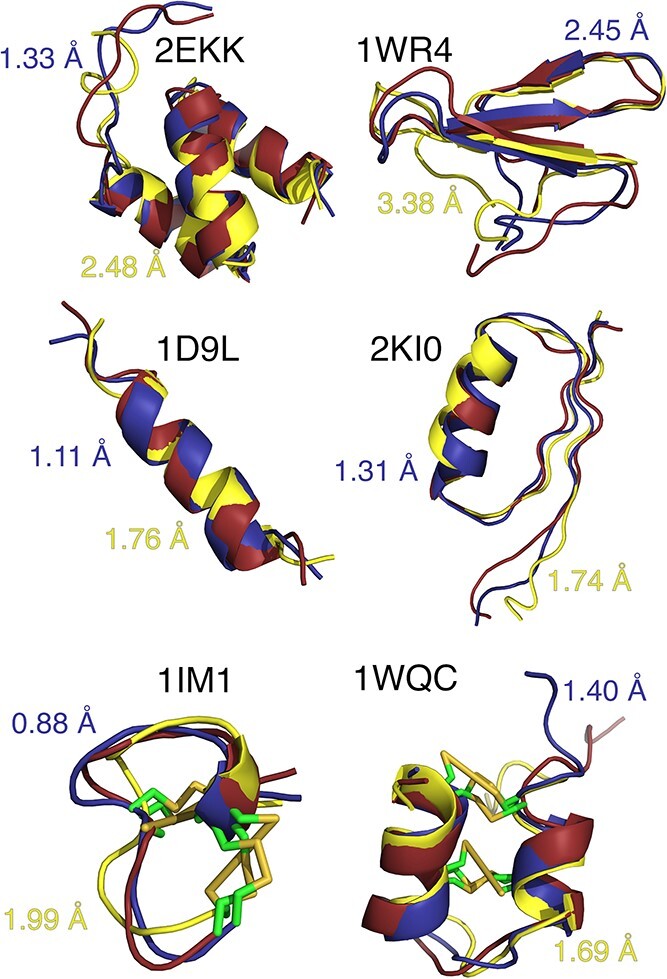
Example CABS-flex predictions (best—blue, prime—yellow) superimposed on the experimental PDB structures (red) using THESEUS [36].

In the medium-length dataset, the averages of the RMSD values for prime structures are the following, in the order of decreasing accuracy: AlphaFold 2.80 Å, ESMFold 3.59 Å, APPTEST 4.49 Å, CABS-flex with contacts 4.81 Å, PEP-FOLD 5.44 Å and CABS-flex 5.94 Å (see Table 1). Interestingly, compared with APPTEST and PEPFOLD, CABS-flex achieved better model quality in the best models category, but the applied ranking and scoring did not allow for their identification as prime models. Medium-length peptides exhibit more propensity to form regular secondary structure than short peptides and consequently less flexibility. AlphaFold, ESMFold and APPTEST neural networks were trained on mostly static structures from PDB and are better equipped to predict fold motifs. Nonetheless, presence of regular secondary structure is beneficial to CABS-flex as well, as seen in examples of 2EKK, 1WR4 and 2KI0 (Figure 2). Moreover, RaptorX contact predictions can provide information on helices and sheets, which is seen in further improvement the quality of prediction in CABS-flex + RaptorX runs.

In the long-length dataset, we observe the same trend as for medium-length peptides. AlphaFold achieves the best accuracy for 18 out of 29 cases with an average RMSD value of 3.62 Å. Next is ESMFold (4.53 Å), with CABS-flex with RaptorX contacts closely behind (4.59 Å). APPTEST is not considered here, because of its limitation of maximum 40 AA. When it comes to best models, CABS-flex with RaptorX contacts achieves the average RMSD value of 3.56 Å, which is close to AlphaFold’s prime average. This again highlights the potential for the method enhancement through ranking and scoring improvement.

### Structure prediction of cyclic peptides

**Figure 3:**
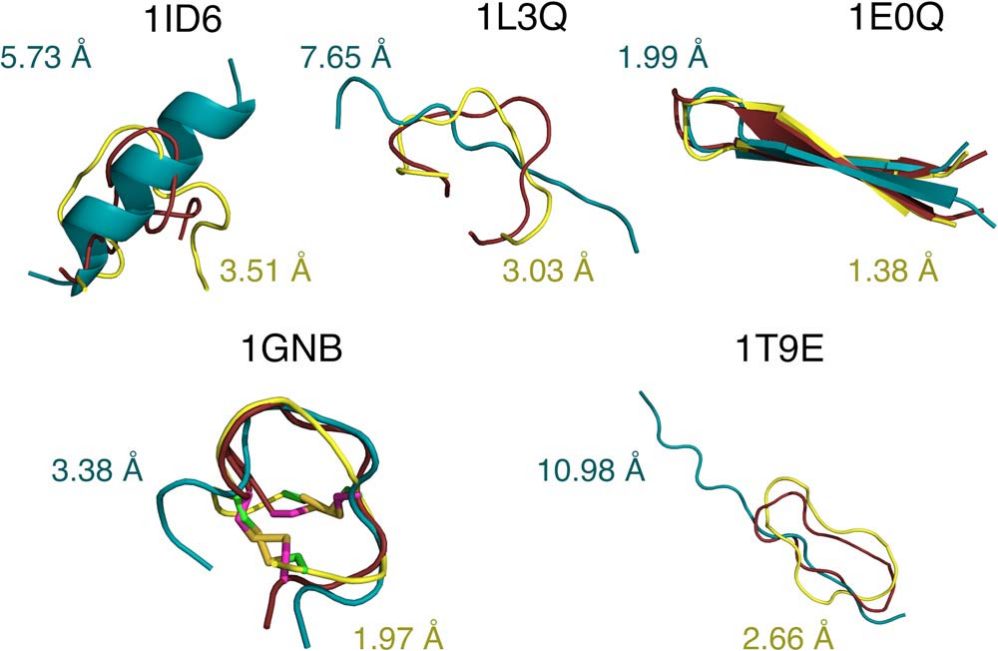
Example CABS-flex (yellow) and AlphaFold (cyan) prime predictions compared with experimental PDB structures (red). The figure shows cases where CABS-flex models were more accurate than AlphaFold models. The models are superimposed using THESEUS [36].

Tables S11 and S12 of Supplementary Materials contain the results for every peptide with disulfide and backbone cyclization, respectively. In the disulfide cyclic dataset, the average of the RMSD values of 1.96 Å indicates a better performance by AlphaFold, as compared with, in the order of decreasing accuracy: ESMFold 2.41 Å, APPTEST 2.67 Å, CABS-flex, 3.04 Å and PEP-FOLD 4.17 Å. While taking into account individual peptide structures, AlphaFold returns a model closest to the native structure for 18 of the 34 peptides in the benchmark, with the remaining 16 structures divided between four other methods. Although AlphaFold does not get explicit information on disulfide bonds, it is capable of extracting this information from MSA, as disulfide bonds are often evolutionarily conserved [36]. There are however examples such as 1GNB (Figure 3) that illustrates the example of AlphaFold prediction where distances between two cysteines exceed the required length for a disulfide bridge. Looking at the structure predicted by CABS-flex, we can see that distance restraints on side chains work reasonably well and allow for rebuilding full-atom models with disulfide bonds present. Other examples of that can be seen in peptides 1IM1 and 1WQC in Figure 2. Considering best models, the average of the RMSD values for CABS-flex is 1.88 Å as compared with the 1.99 and 2.70 Å by APPTEST and PEP-FOLD, respectively. Again, these results suggest that there is room for improvement by using a more efficient scoring function.

For the backbone dataset, APPTEST performs the best, with the average of the RMSD value of 1.57 Å, whereas the second-best method, AlphaFold, achieves a value of 2.14 Å. APPTEST also achieves best quality of prediction in 14 out of 28 peptides, while AlphaFold in 13 out of 28 cases. These results are not surprising because the majority of the backbone dataset is composed of closely related cyclotide peptides, and some of them were used in the training sets. Hence, the comparison may be biased in favor of the deep learning methods, and unfortunately, a small number of experimentally solved backbone cyclic structures hinder proper testing. Lower efficiency of CABS-flex may also be partially due to the specificity of the algorithm that forces the usually linear peptide chain to be closed by distance constraints. The analysis of CABS-flex trajectories showed that in most conformations the distance between C-alpha of first and last residues has the confidence interval between 5.8 and 11.4 Å. This too large gap is corrected at the reconstruction stage by the Modeller algorithm. There is however a single case in which CABS-flex performed the best (see 1T9E in Figure 3). In this case, the AlphaFold prediction did not yield a structure that resembled a ring, while the inclusion of information about backbone cyclization notably enhanced CABS-flex's accuracy in predicting a correct structure.

The field of predicting the structure of cyclic peptides is developing rapidly, lately, as evidenced by very recent publications that delve into various modifications of AlphaFold: AfCycDesign [37] and HighFold [38]. Both of these protocols integrate AlphaFold with additional information regarding backbone cyclization. In addition, HighFold enumerates all possible disulfide bridge pairs enabling a more reliable prediction of disulfide bridge structures. These modifications allowed for further enhancement of the AlphaFold predictive accuracy in the category of backbone cyclized peptides (comparison of AfCycDesign, HighFold and MODPEP2.0 [39] to the methods discussed in this work is presented in the Table S13 of the Supplementary Materials).

### CABS-flex with contact predictions

Providing CABS-flex with contact information, predicted by RaptorX, can significantly improve the quality of predictions (see Table 1). The average of the RMSD values for the best model improves by 0.46 and 0.45 Å for medium and long linear peptides, respectively, and by 0.44 Å for backbone cyclic peptides. The difference in prime structures is even more pronounced. The average of the RMSD values, selected by CABS-flex, improves by 0.70 Å for medium and long linear peptides and by 0.77 Å for backbone cyclic peptides. The inclusion of contact information enhances all (10 000 per run) generated models in CABS-flex, resulting in an improved average conformation. Consequently, the prime model selected from this batch exhibits significantly higher quality compared with a run without contact information.

**Figure 4:**
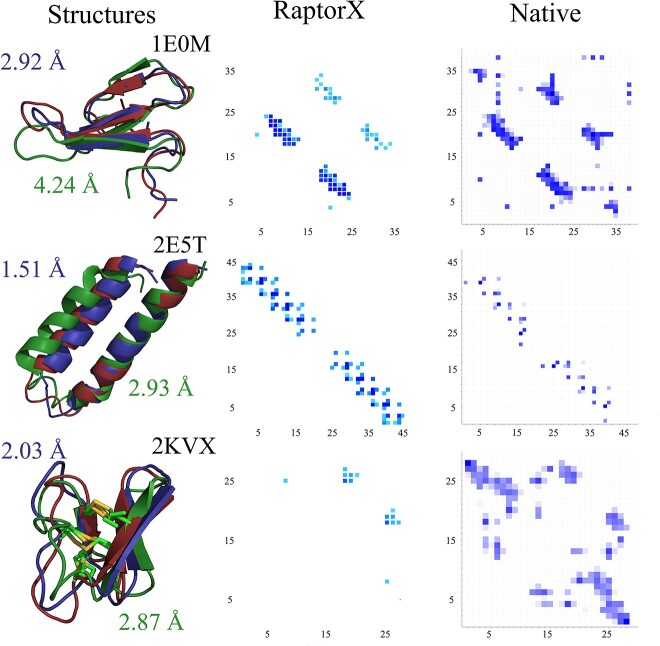
Example contact maps for three peptides—2E0M (top row), 2E5T (middle row), 2KVX (bottom row). The first column contains peptide structures (best from simulation with RaptorX contacts—blue, best from simulation without RaptorX contacts—green) superimposed on the reference state from PDB (red). The second column contains a map of contacts predicted by RaptorX. The third column contains contact maps calculated for native structures, which were generated using web server Mapiya [40].

A comparison between a few of the structures predicted by both methods is shown in Figure 4. Peptides that gained the most by adding information on contacts are typically characterized by regular secondary structure. Contact maps predicted by RaptorX for peptides 1E0M and 2E5T are similar to contact maps calculated for their native reference structures. The analysis of specific patterns confirms that RaptorX successfully predicted the secondary structure of these peptides. In such cases, the quality of structure prediction improves the most. While the secondary structure is sparse for many peptides in our datasets, the inclusion of limited or even individual contact information yields substantial improvements in prediction. For example, RaptorX predicted only a small fraction of native contacts for peptide 2KVX (Figure 4). Despite this limitation, it still improved the quality of the predicted structure by 0.84 Å.

## CONCLUSIONS

We have proposed and evaluated a novel protocol for the *de novo* prediction of linear and cyclic peptide structures. The protocol merges fast simulations in coarse-grained resolution using CABS-flex, with all-atom reconstruction and optimization using Modeller. This work presents the developed extension of the CABS-flex, expanding its applicability to predict structures of both linear and cyclic peptides, including those with backbone and disulfide cyclic bonds. We compared the prediction results with other state-of-the-art algorithms including PEP-FOLD and deep-learning-based methods: APPTEST, ESMfold and AlphaFold. For most of the benchmark cases AlphaFold is clearly superior (as recently also shown in works using AlphaFold tailored for cyclic peptides [37, 38]). CABS-flex is competitive, in particular in the category of short linear peptides. AlphaFold, being a deep learning-based method, has the capacity to capture more intricate and long-range interactions in the peptide sequence. This is clearly seen for longer linear peptides, where the increased complexity in the folding landscape favors AlphaFold’s approach. On the other hand, short peptides can exhibit a wider range of structural behaviors, from disordered regions to well-defined secondary structures. This diversity is not sufficiently represented in experimental structures, which poses challenges in modeling short peptides with deep learning methods. Coarse-grained methods like the CABS model, which rely on simplifications and reduced granularity, can efficiently navigate the restricted conformational space of short peptides and correctly identify dominant structural motifs.

The presented comparison indicates that tested methods can be complementary and their combination into consensus-based prediction protocol may be beneficial. Moreover, we demonstrated that the CABS model has an ability to generate high-quality structures (see dataset of best models in Table 1), which could be identified using a more selective scoring approach e.g. using deep learning. We have also shown that addition of fragmentary contact information (e.g. from predictions) can significantly improve the quality of modeled structures. Furthermore, the presented multiscale modeling pipeline can be easily extended using additional information or different configurations. Other extension possibilities encompass but are not limited to the following options: (1) employing methods such as Molecular Dynamics to investigate peptide dynamics or (2) exploring peptide-based drug design through modifications beyond natural amino acids.

**Key Points**

- We describe a novel CABS-flex-based multiscale protocol for the structure prediction of linear and cyclic peptides.
- We evaluate several state-of-the-art tools; AlphaFold performs the best in most cases, while CABS-flex is competitive for short linear peptides.
- The quality of CABS-flex structure prediction can be enhanced by providing additional structural information, like contact prediction.
- The CABS-flex protocol can be easily extended, or integrated with other peptide structure prediction pipelines.

## Supplementary Material

Supplementary_info_rev2_v2_bbae003

## Data Availability

The data underlying this article will be shared on reasonable request to the corresponding author.

## Author Biographies

**Aleksandra E. Badaczewska-Dawid** is a Research Scientist IV at the Genome Informatics Facility, Iowa State University. Her research interests are bioinformatics and computational biology.

**Karol Wróblewski** is currently a PhD student in The Interdisciplinary Doctoral School, University of Warsaw. His research interests are structural biology and peptide therapeutics.

**Mateusz Kurcinski** is an assistant professor at the University of Warsaw, working in the Laboratory of Computational Biology at Biological and Chemical Research Center.

**Sebastian Kmiecik** is a professor at University of Warsaw, head of Laboratory of Computational Biology at Biological and Chemical Research Centre, University of Warsaw.
