# Correction to: Structure prediction of linear and cyclic peptides using CABS-flex

**DOI:** 10.1093/bib/bbae605

**Published:** 2024-11-15

This is a correction to: Aleksandra Badaczewska-Dawid, Karol Wróblewski, Mateusz Kurcinski, Sebastian Kmiecik, Structure prediction of linear and cyclic peptides using CABS-flex, *Briefings in Bioinformatics*, Volume 25, Issue 2, March 2024, bbae003, https://doi.org/10.1093/bib/bbae003

In the originally published version of this paper, the grant number in the following sentence of the Funding section required correction:

MK acknowledges funding from the National Science Centre [501/D112/66 GR-6271].

The corrected sentence reads:

MK acknowledges funding from the National Science Centre [2019/33/B/NZ2/02100].

