# Supplementary material for: Structure prediction of linear and cyclic peptides using CABS-flex
Source: Brief Bioinform. 2024 Feb 1;25(2):bbae003. doi: 10.1093/bib/bbae003 (PMC10836054; doi:10.1093/bib/bbae003)
Supplement: Supplementary_info_rev2_v2_bbae003 [file supplementary_info_rev2_v2_bbae003.docx]

Supplementary Materials to the manuscript **Structure prediction of linear and cyclic peptides using CABS-flex**

**Table S1.** The study comprised 8 series of simulations with varying input data depending on the dataset type (linear, cyclic) and modeling objectives (with or without predicted contacts). The abbreviations in the right part of the table refer to a series of simulations as follows: LINEAR (S - short, M - medium, M^CON^ - medium with contacts, L - long, L^CON^ - long with contacts) and CYCLIC (D - disulfide, B - backbone, B^CON^ - backbone with contacts). The left part of the table describes the types of inputs used and the definition of each option, accompanied by practical examples. The syntax for distance restraints takes four ordered arguments: identifiers (i, j) for the pair of residues, restraint’s distance, and weight. For example, the restraint for the disulfide bridge between Cys-3 and Cys-8 in chain A is defined as follows: “*3:A 8:A 2.0 1.0”*. That restraint favors during the simulation the spatial contact of specified residues at a distance of 2 Å. If residues distance exceeds the user-defined threshold of 2.0 Å, the energetic penalty linearly increases with the slope defined by the restraint weight 1.0.

|  | |  |  | **LINEAR** | | | | | **CYCLIC** | | |
| --- | --- | --- | --- | --- | --- | --- | --- | --- | --- | --- | --- |
| *INPUT TYPE* | | *OPTION SYNTAX* | | S | M | M^CON^ | L | L^CON^ | D | B | B^CON^ |
| *i* | *AA sequence* | **-i SEQUENCE:SECONDARY_STRUCTURE**  *-i GRCCHPACGKYYSC:CCCCCHHCCCCCCC* | | ⧫ | ⧫ | ⧫ | ⧫ | ⧫ | ⧫ | ⧫ | ⧫ |
| *ii* | *sec. structure* |  |  | ⧫ | ⧫ | ⧫ | ⧫ | ⧫ | ⧫ | ⧫ | ⧫ |
| *iii* | *disulfide restrains* | **--**[**sc-rest-add RESI RESJ DIST WEIGHT**](https://bitbucket.org/lcbio/cabsflex/wiki/Home#markdown-header--sc-rest-add-resi-resj-dist-weight)  *--*[*sc-rest-add*](https://bitbucket.org/lcbio/cabsflex/wiki/Home#markdown-header--sc-rest-add-resi-resj-dist-weight) *3:A 8:A 2.0 1.0*  or  **--sc-rest-file FILE**  *3:A 8:A 2.0 1.0 # first disulfide bridge*  *4:A 14:A 2.0 1.0 # second restraint* | |  |  |  |  |  | ⧫ | ⧫* | ⧫* |
| *iv* | *backbone restraints* | **--ca**[**-rest-add RESI RESJ DIST WEIGHT**](https://bitbucket.org/lcbio/cabsflex/wiki/Home#markdown-header--sc-rest-add-resi-resj-dist-weight)  *--ca*[*-rest-add*](https://bitbucket.org/lcbio/cabsflex/wiki/Home#markdown-header--sc-rest-add-resi-resj-dist-weight) *1:A 14:A 3.8 1.0*  or  **--ca-rest-file FILE**  *1:A 14:A 3.8 1.0 # backbone cyclization*  *1:A 10:A 6.0 0.75 # first contact*  *2:A 12:A 6.0 0.75 # second contact*  *6:A 12:A 6.0 0.75 # third contact, etc.* | |  |  |  |  |  |  | ⧫ | ⧫ |
| *v* | *contacts restraints* |  |  |  |  | ⧫ |  | ⧫ |  |  | ⧫ |

*Disulfide restraints are only used if the modeled peptide has at least one disulfide bridge.

**Table S2.** CABS-flex settings and coarse-grained (CG) results for the optimization simulations of the peptide folding protocol. The *-a* option determines the number of MC annealing cycles; by default, it is 20, and once set to 1, it switches the simulation to isothermal mode. This value multiplied by the number of great Monte Carlo cycles determines the simulation time, so to keep it at the same level, the value of the parameter *-y* must be increased accordingly. Parameter *-D* regulates the temperature increment between replicas by increasing the initial energy scaling factor for consecutive replicas by this value. Thus, if the first replica starts with a temperature factor of 3 at an increment of 0.1, then 3.9 is an initial temperature for replica 10, and at an increment of 0.5, it will be 7.5. The row for the *-t* option shows the range of reduced temperature for the first replica, and the corresponding range for the 10th replica is in the parenthesis. The last two lines show the average modeling performance over the linear LONG dataset measured as the BCscore and CA-RMSD (in the parenthesis) calculated on the CG results of CABS-flex modeling.

|  | **RUN 1** | RUN 2 | RUN 3 | RUN 4 | RUN 5 | **RUN6** | RUN 7 | RUN 8 |
| --- | --- | --- | --- | --- | --- | --- | --- | --- |
| type | Ann | Ann | Ann | IsoT | IsoT | IsoT | IsoT | IsoT |
| -a | 20 | 20 | 20 | 1 | 1 | 1 | 1 | 1 |
| -y | 50 | 50 | 50 | 1000 | 1000 | 1000 | 1000 | 1000 |
| -D | 0.1 | 0.2 | 0.5 | 0.1 | 0.15 | 0.2 | 0.25 | 0.3 |
| -t | 3.0 - 1.0  (3.9-1.9) | 3.0 - 1.0  (4.8-2.8) | 3.0 - 1.0  (7.5-5.5) | 1.0  (1.9) | 1.0  (2.35) | 1.0  (2.8) | 1.0  (3.25) | 1.0  (3.7) |
| PRIME | **0.64**  (4.61) | 0.62  (4.60) | 0.58  (5.22) | 0.41  (6.17) | 0.54  (5.12) | **0.65**  (4.44) | 0.60  (4.75) | 0.61  (4.73) |
| BEST | **0.83**  (3.46) | 0.81  (3.37) | 0.80  (4.22) | 0.61  (5.49) | 0.77  (4.07) | **0.82**  (3.50) | 0.81  (3.65) | 0.80  (3.82) |

**Simulation parameters**

For all the simulation series in Table S1, we applied Monte Carlo (MC) with Replica Exchange schema for sampling the conformational space. Each computational experiment was composed of 10 replicas distributed evenly along with the energy scaling factor. To optimize the modeling settings for the proposed peptide folding protocol, we performed systematized tests by tuning (i) the ranges of reduced temperature [*-t* option], (ii) temperature increment between replicas [*-D* option], and (iii) number of MC annealing cycles [*-a* option] (i.e., annealing vs. isothermal variants). The preliminary tests were executed on linear peptides from the LONG dataset. Table S2 summarizes the optimization settings and coarse-grained results (BCscore and CA-RMSD) averaged over the dataset. As shown in Table S2, test runs 1 and 6 provide comparably high performance, with their settings considered optimal for annealing (Ann) and isothermal (IsoT) modeling schema, respectively. In this work, we proceeded further with the settings for the annealing mode (run 1).

**Structural information**

Importantly, the amino acid sequence and secondary structure assignment are entered together with option *-i* (see Table S1). If no secondary structure is provided, CABS-flex will use PsiPred by default to predict it from the sequence. Regardless of their nature, distance restraints can be applied either to alpha carbons (--ca) or coarse-grained (--sc) pseudo-atoms imitating the amino acid side chain. A single restraint is specified on the command line with the *--xx-rest-add* flag, while more numerous can be grouped in a text file and loaded with the *--xx-rest-file* option. In particular, we imposed distance restraints on side groups of cysteine pairs known to form the disulfide bridge. By contrast, cyclization through the backbone ends and all the predicted intramolecular contacts were kept solely on the alpha carbons of residue pairs. The distance cutoffs of 3.8 Å and 6.0 Å were assumed for a backbone peptide bond and intramolecular contacts, respectively.

**All-atom reconstruction**

In this work, for each peptide target, we rebuild sets of 10 top-scored models (prime models) and 100 best models. The prime model is selected from the rebuilt top10 subset by the lowest AA-RMSD relative to the known experimental structure. To find the best model out of 10,000, we rebuilt a set of 100 models with lowest CA-RMSD referenced to the known experimental structures. We opted to rebuild not just the top-ranked model but the larger number like 100 models to ensure the high quality of the local peptide geometry. This decision was motivated by the observation of occasional artifacts arising from the utilization of the CABS, i.e., coarse-grained and lattice-based model. These unphysical distortions could manifest as reconstructed structures with local steric clashes, leading to a lower all-atom quality than indicated by the CA-based evaluation.

**Experimental reference structures**

Most of the peptides benchmarked in this study have a known experimental structure determined by NMR spectroscopy. Thus, the dynamic nature of the structure for flexible peptides is encoded by average in the bundle of 5-20 conformations. The reference state, used solely for assessing the modeling efficiency, was selected as a PDB model with the lowest RMSD or highest BCscore calculated relative to the top-scored model from the simulation trajectory. For the remaining cases with known crystallographic structures, the only available model was used as the reference.

The Tables from S3 to S7 contain the same columns, which are from left to right:

- PDB - This is a pdb code.
- SEQUENCE : SEC. STRUCTURE - This is a string used as input. The first part consists of sequence extracted from "SEQRES” records of the PDB files deposited in the Protein Data Bank. The second part consists of the secondary structure assigned by the DSSP algorithm from the experimental structure.
- LEN - This is the number of residues.
- RIGID CORE - This is a definition of which residues constitute a rigid core. 2-11:16-33 means that all the residues from 2 to 11, included, and from 16 to 33, included, are considered the rigid core.
- NMR No. - This is the number of models contained in the PDB file.
- NATIVE CONTACTS - Native contacts were identified from known PDB structures using an in-house method that filters for residue pairs at least 6 positions apart (i - i+6) and having alpha atoms within 8.5 Angstroms. 1-11 means that there is a contact between amino acids 1 and 11.
- Raptor-X CONTACTS - These are contacts predicted by the RaptorX method. They are used as distance restraints in simulations indicated by “**+ RaptorX contacts**”.

**Table S3.** Dataset 1: short linear peptides

| PDB | SEQUENCE : SEC. STRUCTURE | LEN | RIGID CORE | NMR No. | NATIVE CONTACTS | Raptor-X CONTACTS |
| --- | --- | --- | --- | --- | --- | --- |
| 1A13 | INWKGIAAMAKKLL:CCCCCHHHHHCCCC | 14 | 2-14 | 14 |  |  |
| 1B03 | RKSIRIQRGPGRAFVTIG:CEEEEECCCCCCEEEEEC | 18 | - | 35 |  |  |
| 1C98 | GNLWATGHFM:CCCCHHHHCC | 10 | - | 20 |  |  |
| 1D6X | VRRFPWWWPFLRR:CCCCCCCHHHCCC | 13 | 1-12 | 19 |  |  |
| 1D7N | INLKALAALAKKIL:CCCHHHHHHHHCCC | 14 | 2-13 | 10 |  |  |
| 1D9J | KWKLFKKIGIGKFLHSAKKF:CCHHHHHCCCCCCCCHHHCC | 20 | 10-20 | 20 |  |  |
| 1D9L | KWKLFKKIKFLHSAKKF:CCCCHHHHHHHHHCCCC | 17 | 4-15 | 20 |  |  |
| 1D9M | KWKLFKKIPKFLHSAKKF:CCHHHHCCCCHHHHHCCC | 18 | 10-18 | 20 |  |  |
| 1D9O | KAKLFKKIGIGKFLHSAKKF:CCCCCCCCCCCHHHHHHHHC | 20 | 11-20 | 20 |  |  |
| 1D9P | KLKLFKKIGIGKFLHSAKKF:CCCHHHHHCCHHHHHHHHCC | 20 | 10-20 | 20 |  |  |
| 1DN3 | QAPAYKKAAKKLAES:CCCHHHHHHHHHHCC | 15 | - | 1 |  |  |
| 1DU1 | TSAQKAKAEERKRRKMSRGL:CHHHHHHCCCCCCCCCCCCC | 20 | - | 1 |  |  |
| 1E0Q | MQIFVKTLDGKTITLEV:CEEEEEECCCEEEEEEC | 17 | - | 27 |  |  |
| 1EGS | TKSAGGIVL:CCCCCCCCC | 9 | - | 20 |  |  |
| 1G89 | ILPWKWPWWPWRR:CCCCCCCCCCCCC | 13 | 1-12 | 16 |  |  |
| 1GJF | RAGPLQWLAEKYQG:CCCCCHHHHHHHHC | 14 | 4-14 | 1 |  |  |
| 1HU5 | KNLRRIIRKIIHIIKKYG:CCCHHHHHHHHHHHHHCC | 18 | 2-18 | 20 |  |  |
| 1HU6 | KNLRRIIRKGIHIIKKYG:CCCHHHHHHHHCCHHHCC | 18 | 2-18 | 20 |  |  |
| 1HU7 | KNLRRITRKIIHIIKKYG:CCCCCCHHHHHHHHHHHC | 18 | 2-18 | 20 |  |  |
| 1ID6 | SVQARWEAAFDLDLY:CCCCCCCCCCCCCCC | 15 | 1-14 | 11 |  |  |
| 1IN3 | RPLQWLAEKYFQ:CCCHHHHHHCCC | 12 | - | 20 |  |  |
| 1JAV | KWASLWNWFNITNWLWYIK:CHHHCHHHHCHHHHHHHHC | 19 | - | 1 |  |  |
| 1KZV | FPRIWLHNLGQHIYETYG:CCCHHHHHHHHHHHHHHC | 18 | 2-18 | 20 |  |  |
| 1L2Y | NLYIQWLKDGGPSSGRPPPS:CHHHHHHHCCHHHHCCCCCC | 20 | - | 38 |  |  |
| 1L3Q | FPGKNVNCTSGE:CCCCCCCCCCCC | 12 | - | 1 |  |  |
| 1LCX | ELLELDKWASLWN:CCCHHHHHHHHHC | 13 | 3-12 | 25 |  |  |
| 1M02 | HPLKQYWWRPSI:CCCCCCCCCCCC | 12 | 2-11 | 19 |  |  |
| 1MYU | DVPKSDQFVGLM:CCCHHHHHHHHC | 12 | - | 20 |  |  |
| 1NIZ | KRIHIGPGRAFYTT:CEEEECCCCEEEEC | 14 | - | 1 |  |  |
| 1NKF | DKDGDGYISAAEAAAQ:CCCCCCCCCHHHHHHC | 16 | - | 30 |  |  |
| 1ODP | YSDELRQRLAARLEALKENG:CCHHHHHHHHHHHHHHHCCC | 20 | - | 5 |  |  |
| 1P0J | AKKVFKRLEKLFSKIQNWK:CCCCHHHHHHHHHHHHHHC | 19 | 2-19 | 20 |  |  |
| 1P0L | AKKVFKRLEKLFSKIWNDK:CCCCHHHHHHHHHHHHHHC | 19 | 2-19 | 20 |  |  |
| 1P0O | AKKVFKRLEKLFSKIWNWK:CCCCHHHHHHHHHHHHHHC | 19 | 2-19 | 20 |  |  |
| 1P5K | AKKVFKRLEKSFSKIQNDK:CCCCHHHHHHHHHHHHHCC | 19 | 2-18 | 20 |  |  |
| 1PEF | EQLLKALEFLLKELLEKL:CHHHHHHHHHHHHHHCCC | 18 | - | 1 |  |  |
| 1QCM | GSNKGAIIGLM:CCCHHHHHHHC | 11 | 2-11 | 20 |  |  |
| 1QFA | LRHYLNLLTRQRY:CHHHHHHHHHHHC | 13 | - | 20 |  |  |
| 1RPV | TRQARRNRARRWRARQR:CCCHHHHHHHHHHHCCC | 17 | 4-16 | 20 |  |  |
| 1SOL | KHVVPNEVVVQRLFQVKGRR:CCCCCCCCCHHHHHHHHCCC | 20 | - | 1 |  |  |
| 2BP4 | DAEFRHDSGYEVHHQK:CHHHHHHHHHHHHHCC | 16 | 1-15 | 20 |  |  |
| 2BTA | MEELQDDYEDMMEEN:CCCCCCCCCCCCCCC | 15 | 4-9 | 1 |  |  |

**Table S4.** Dataset 2: medium linear peptides

| PDB | SEQUENCE :  SEC. STRUCTURE | LEN | RIGID CORE | NMR No. | NATIVE CONTACTS | Raptor-X CONTACTS |
| --- | --- | --- | --- | --- | --- | --- |
| 1BHI | MSDDKPFLCTAPGCGQRFTNEDHLAVHKHKHEMTLKFG:CCCCCCEECCCCCCCCEECCHHHHHHHHHHHHCCCCCC | 38 | 7-11:14-33 | 20 | 4-19, 5-19, 6-17, 6-18, 6-19, 6-20, 7-17, 7-18, 7-19, 7-20, 7-21, 7-24, 8-15, 8-16, 8-17, 8-18, 8-24, 9-15, 9-16, 9-17, 9-24, 9-27, 9-28, 10-28, 11-28, 11-31, 17-24, 18-24, 30-36 | 7-18, 8-17, 6-17, 6-19, 7-17, 6-18, 9-18, 7-24, 9-16, 8-16, 5-19, 8-15, 9-24, 9-15, 18-24, 7-19, 7-21, 7-20, 18-27, 9-27, 14-27, 9-28, 4-19, 9-17, 8-18, 14-31, 11-28, 3-19, 11-31, 5-20, 13-31, 14-28, 9-31 |
| 1BWX | SVSEIQLMHNLGKHLNSMERVEWLRKKLQDVHNFVALGA:CCCCHHHHHHCCCCCCCHHHHHHHHHHHHHHHCCCCCCC | 39 | 15-28 | 10 | 7-14, 7-15, 7-16, 8-19, 11-18, 11-19, 12-19, 12-20, 12-22, 12-23, 13-19, 14-20, 15-21, 15-23, 28-39, 29-39, 31-37, 31-39, 32-38, 32-39 | 2-31, 5-27, 5-31 |
| 1BY0 | RKKLEELERDLRKLKKKIKKLEEDNPW:CCHHHHHHHHHHHHHHHHHHHCCCCCC | 27 | 1-23 | 20 |  |  |
| 1E0L | GSMGATAVSEWTEYKTADGKTYYYNNRTLESTWEKPQELK:CCCCCCCCCCCEEEECCCCCEEEEECCCCEEECCCCCCCC | 40 | 9-40 | 10 | 3-35, 4-35, 5-35, 5-36, 5-37, 6-35, 6-36, 7-22, 7-23, 7-24, 8-20, 8-21, 8-22, 8-23, 8-36, 9-20, 9-21, 9-22, 9-23, 10-18, 10-19, 10-20, 10-21, 11-18, 11-19, 11-20, 11-21, 12-18, 12-19, 13-19, 18-30, 19-28, 19-29, 19-30, 19-31, 19-32, 20-28, 20-29, 20-30, 20-31, 20-32, 21-27, 21-28, 21-29, 22-28 | 11-23, 12-24, 11-25, 10-26, 13-23, 11-24, 14-22, 12-26, 14-24, 10-25, 10-27, 12-23, 13-21, 15-21, 11-26, 13-22, 14-21, 16-22, 14-23, 24-31, 22-33, 15-22, 13-24, 9-26, 22-31, 23-31, 25-31, 23-32, 23-33, 24-30, 20-33, 9-27, 12-25 |
| 1E0M | SMGLPPGWDEYKTHNGKTYYYNHNTKTSTWTDPRMSS:CCCCCCCEEEEECCCCCEEEEECCCCEEECCCCCCCC | 37 | 3-35 | 10 | 3-34, 4-33, 4-34, 5-33, 5-34, 7-21, 7-22, 7-23, 7-24, 8-20, 8-21, 8-22, 8-23, 8-33, 9-20, 9-21, 9-22, 10-18, 10-19, 10-20, 10-21, 11-17, 11-18, 11-19, 11-20, 11-21, 12-18, 12-19, 13-19, 17-30, 18-30, 19-28, 19-29, 19-30, 19-31, 19-32, 20-28, 20-29, 20-30, 20-31, 20-32, 20-33, 21-27, 21-28, 21-29, 22-28, 29-37, 31-37 | 7-23, 8-20, 9-21, 8-22, 10-20, 8-21, 11-19, 9-23, 7-22, 7-24, 8-23, 9-20, 12-18, 11-21, 10-18, 10-19, 11-18, 13-19, 21-28, 19-30, 11-20, 12-19, 10-21, 17-30, 19-28, 20-28, 22-28, 6-24, 21-27, 20-29, 4-20, 18-33, 17-32, 9-22, 20-30, 18-31 |
| 1E0N | MGGRLPPGWEIIHENGRPLYYNAEQKTKLHYPPSGSS:CCCCCCCCEEEEECCCCEEEEECCCCEEECCCCCCCC | 37 | 7-31 | 10 | 1-18, 2-16, 2-17, 2-18, 2-19, 3-14, 3-15, 3-16, 3-17, 3-18, 3-21, 4-14, 4-15, 4-16, 5-12, 5-13, 5-14, 5-15, 6-12, 6-13, 6-14, 6-15, 7-13, 12-24, 13-22, 13-23, 13-24, 13-25, 14-22, 14-23, 14-24, 14-25, 15-21, 15-22, 15-23, 16-22 | 12-19, 9-21, 8-23, 9-20, 9-22, 10-21, 8-22, 11-20, 12-21, 9-23, 11-19, 10-23, 10-20, 11-18, 12-18, 8-24, 12-20, 13-19, 7-23 |
| 1I6C | KLPPGWEKRMSRSSGRVYYFNHITNASQWERPSGNSSSG:CCCCCEEEEECCCCCCEEEEECCCCEEECCCCCCCCCCC | 39 | 2-11:16-33 | 10 | 1-21, 1-28, 1-30, 1-32, 2-19, 2-20, 2-21, 2-26, 2-32, 3-21, 3-32, 4-21, 4-22, 5-21, 5-22, 5-23, 6-19, 6-20, 6-21, 6-22, 7-19, 7-20, 7-21, 7-22, 8-17, 8-18, 8-19, 8-20, 8-31, 9-17, 9-18, 9-19, 9-20, 10-16, 10-17, 10-18, 11-17, 11-18, 17-29, 18-27, 18-28, 18-29, 18-30, 19-27, 19-28, 19-29, 19-30, 19-31, 20-26, 20-27, 20-28, 21-27, 33-39 | 6-19, 7-20, 6-21, 5-22, 8-19, 9-18, 6-20, 5-21, 7-22, 9-20, 10-17, 8-17, 7-19, 5-23, 6-22, 8-18, 9-17, 18-29, 20-27, 10-16, 11-18, 9-19, 16-29, 10-18, 18-27, 19-27, 20-26, 8-20, 19-28, 4-23, 21-27, 11-17, 2-19, 18-28, 17-30, 19-29, 16-31, 17-32, 17-29, 7-21, 19-30 |
| 1JRJ | HGEGTFTSDLSKQMEEEAVRLFIEWLKNGGPSSGAPPPS:CCCCCCHHHHHHHHHHHHHHHHHHHHHCCHHHCCCCCCC | 39 | 11-38 | 36 | 18-38, 18-39, 21-38, 22-37, 22-38, 22-39, 25-37, 25-38, 30-36, 30-37, 31-37 | 6-14, 25-31 |
| 1WR3 | GSPPLPPGWEEKVDNLGRTYYVNHNNRSTQWHRPSL:CCCCCCCCEEEEECCCCCEEEEECCCCCEECCCCCC | 36 | 5-15:17-34 | 20 | 5-34, 5-35, 8-23, 8-24, 8-25, 9-21, 9-22, 9-23, 9-24, 10-21, 10-22, 10-23, 10-24, 11-19, 11-20, 11-21, 11-22, 12-19, 12-20, 12-21, 12-22, 13-19, 13-20, 14-20, 18-31, 19-31, 20-29, 20-30, 20-31, 20-32, 20-33, 21-28, 21-29, 21-30, 21-31, 21-32, 21-33, 21-34, 22-28, 22-29, 22-30, 23-29 | 9-21, 10-22, 9-23, 8-24, 11-21, 9-22, 12-20, 8-23, 10-24, 12-22, 10-21, 9-24, 13-19, 8-25, 11-19, 11-20, 12-19, 14-20, 22-29, 12-21, 20-31, 13-20, 11-22, 5-21, 20-29, 18-31, 7-25, 21-29, 23-29, 22-28, 10-23, 21-30 |
| 1WR4 | GSPGLPSGWEERKDAKGRTYYVNHNNRTTTWTRPIM:CCCCCCCCEEEEECCCCCEEEEECCCCEEECCCCCC | 36 | 5-34 | 20 | 1-11, 2-11, 4-34, 5-33, 5-34, 5-35, 5-36, 6-34, 6-35, 6-36, 7-25, 8-23, 8-24, 8-25, 8-26, 9-21, 9-22, 9-23, 9-24, 9-25, 9-34, 10-21, 10-22, 10-23, 10-24, 11-19, 11-20, 11-21, 11-22, 12-19, 12-20, 12-21, 12-22, 13-19, 13-20, 14-20, 19-31, 20-29, 20-30, 20-31, 20-32, 21-29, 21-30, 21-31, 21-32, 21-33, 21-34, 22-28, 22-29, 22-30, 23-29, 23-30 | 9-21, 10-22, 8-24, 9-23, 9-22, 11-21, 12-20, 8-23, 10-24, 12-22, 8-25, 9-24, 10-21, 13-19, 11-19, 11-20, 12-19, 14-20, 22-29, 12-21, 20-31, 13-20, 11-22, 7-25, 5-21, 20-29, 18-31, 23-29, 21-29, 7-24, 21-30, 10-23, 22-28 |
| 1WY3 | LSDEDFKAVFGMTRSAFANLPLWLQQHLKKEKGLF:CCHHHHHHHHCCCHHHHHHCCHHHHHHHHHHHCCC | 35 | 1-35 | 1 | 3-13, 3-14, 6-12, 6-13, 6-14, 7-13, 7-14, 9-32, 10-28, 10-32, 17-25, 18-25, 29-35 | 3-14, 6-17, 6-14, 7-14, 6-12, 9-32, 10-32, 7-13, 10-17, 10-28, 9-34, 20-28 |
| 1YIU | GAMGPLPPGWEKRTDSNGRVYFVNHNTRITQWEDPRS:CCCCCCCCCCCEEECCCCCEEEEECCCCEEECCCCCC | 37 | 5-36 | 8 | 3-36, 3-37, 4-36, 5-36, 6-12, 6-35, 6-36, 7-35, 7-36, 9-23, 9-24, 9-25, 9-26, 10-22, 10-23, 10-24, 10-25, 11-22, 11-23, 11-24, 12-20, 12-21, 12-22, 12-23, 13-20, 13-21, 13-22, 13-23, 14-20, 14-21, 15-21, 20-32, 21-30, 21-31, 21-32, 21-33, 21-34, 22-30, 22-31, 22-32, 22-33, 22-34, 22-35, 23-29, 23-30, 23-31, 24-30, 24-31 | 10-22, 11-23, 10-24, 9-25, 12-22, 10-23, 13-21, 9-24, 11-25, 13-23, 10-25, 9-26, 11-22, 12-20, 14-20, 12-21, 13-20, 15-21, 21-32, 23-30, 13-22, 14-21, 19-32, 12-23, 21-30, 8-26, 22-30, 24-30, 6-22, 23-29, 22-31, 20-33, 19-34, 11-24, 22-32, 20-32 |
| 1YYB | GSADEELEALRRQRLAELQAKHGDPGD:CCHHHHHHHHHHHHHHHHHHCCCCCCC | 27 | 2-21 | 20 |  |  |
| 2BN6 | GADYSAQWAEYYRSVGKIEEAEAIEKTLKNKQN:CCCCCCHHHHHHCCCCCHHHHHHHHHCCCCCCC | 33 | 4-29 | 29 | 1-30, 1-31, 2-28, 2-30, 2-31, 3-28, 3-29, 3-30, 4-27, 4-28, 5-24, 5-25, 5-26, 5-27, 5-28, 5-29, 6-24, 6-25, 6-26, 6-27, 6-28, 8-24, 8-25, 9-18, 9-21, 9-22, 9-24, 9-25, 10-18, 10-21, 11-17, 11-18, 12-18, 12-19, 12-20, 12-21, 13-19, 13-21 | 9-24, 9-25, 12-21, 9-21, 5-28, 13-21, 9-28, 6-28, 12-24, 8-24, 5-24, 12-20, 12-18, 2-28, 2-31, 5-27, 5-31, 2-32 |
| 2GDL | LVQRGRFGRFLRKIRRFRPKVTITIQGSARF:CCCCHHHHCCCCCCCCCCCCCHHHHHHHHHC | 31 | 8-8:10-11:14-15:21-29 | 9 | 9-15, 14-22, 14-23, 15-21, 15-22, 15-23, 16-22, 16-23, 16-24 |  |
| 2K76 | PFPPTPPGEEAPVEDLIRFYNDLQQYLNVV:CCCCCCCCCCCCHHHHHHHHHHHHHHHHHC | 30 | 4-29 | 10 | 2-26, 4-19, 4-22, 4-23, 5-19, 6-16, 6-19, 7-15, 7-16, 7-19 | 11-19 |
| 2KBL | GSGYIPRAPRDGQAYVRKDGEWVLLSTFL:CCCCCCCCCCCCCCEEEECCEEEECCCCC | 29 | 8-29 | 10 | 1-17, 1-18, 2-18, 3-18, 3-19, 4-18, 4-19, 4-20, 6-13, 6-20, 7-13, 11-22, 11-23, 11-24, 12-21, 12-22, 12-23, 12-24, 13-20, 13-21, 13-22, 13-23, 14-20, 14-21, 14-22, 14-23, 15-21 | 15-22, 14-25, 16-25, 15-24, 16-23, 15-23, 15-25, 16-22, 13-24, 14-24, 14-26, 17-23, 14-27, 13-26, 16-24, 14-28 |
| 2KI0 | GSGQVRTIWVGGTPEELKKLKEEAKKANIRVTFWGD:CCCCCCCCCCCCCHHHHHHHHHHHHHHCCCCCCCCC | 36 | 5-11:13-36 | 20 | 4-27, 4-29, 5-28, 5-29, 5-30, 6-29, 6-30, 7-29, 7-30, 7-31, 7-32, 8-30, 8-31, 8-32, 8-33, 9-31, 9-32, 9-33, 9-34, 10-16, 10-32, 10-33, 10-34, 10-36, 11-33, 11-34, 11-35, 11-36, 12-33, 12-34, 12-35, 12-36, 17-33, 20-31, 21-31, 23-29, 24-30, 24-31 | 8-31, 9-32, 6-29, 8-32, 7-30, 10-34, 7-32, 11-34, 9-34, 6-30, 11-35, 10-33, 9-33, 12-35, 7-29, 9-31, 5-29, 7-31, 8-29, 11-33, 5-30, 11-36, 12-36 |
| 2KYA | MNKKNILPQQGQPVIRLTAGQLSSQLAELSEEAL:CCCCCCCCCCCCHHHHHHHHHHHHHHHCCCCCCC | 34 | 11-30 | 14 |  | 17-23, 17-26 |
| 2L0G | GHMFPSDIDPQVFYELPEAVQKELLAEWKRTG:CCCCCCCCCHHHHHHCCHHHHHHHHHHHHHHC | 32 | 5-32 | 20 | 1-7, 1-8, 1-9, 1-10, 2-8, 2-9, 2-10, 3-9, 3-10, 4-10, 13-21, 14-21 | 13-21, 16-24, 13-24, 8-24, 8-28, 5-28 |
| 2OVC | GAVDEISMMGRVVKVEKQVQSIEHKLDLLLGFY:CCCCHHHHHHHHHHHHHHHHHHHHHHHHHHCCC | 33 | 4-33 | 1 | 24-30 |  |
| 2YSC | GSSGSSGGLPPGWRKIHDAAGTYYWHVPSGSTQWQRPTW:CCCCCCCCCCCCEEEEEECCEEEEEECCCCCEECCCCCC | 39 | 8-39 | 20 | 1-7, 7-38, 8-38, 8-39, 9-36, 9-37, 9-38, 9-39, 10-38, 10-39, 12-26, 12-27, 12-28, 12-29, 13-24, 13-25, 13-26, 13-27, 13-28, 14-24, 14-25, 14-26, 14-27, 15-22, 15-23, 15-24, 15-25, 16-22, 16-23, 16-24, 16-25, 17-23, 22-34, 23-32, 23-33, 23-34, 23-35, 23-36, 24-32, 24-33, 24-34, 24-35, 24-36, 24-37, 25-31, 25-32, 25-33, 26-32 | 13-24, 13-26, 12-27, 14-25, 13-25, 14-27, 15-24, 16-23, 12-26, 16-25, 13-27, 12-28, 14-24, 15-22, 15-23, 16-22, 16-24, 25-32, 23-34, 17-23, 9-24, 26-32, 15-25, 11-28, 26-33, 23-32, 24-34 |
| 2YSF | GSSGSSGLPEGWEMRFTVDGIPYFVDHNRRTTTYIDPRTG:CCCCCCCCCCCEEEEECCCCCEEEEECCCCCEECCCCCCC | 40 | 7-39 | 20 | 5-14, 8-14, 8-37, 8-38, 11-25, 11-26, 11-27, 11-28, 11-29, 12-24, 12-25, 12-26, 12-27, 12-37, 13-24, 13-25, 13-26, 13-27, 14-22, 14-23, 14-24, 14-25, 15-22, 15-23, 15-24, 15-25, 16-22, 16-23, 16-24, 17-23, 22-34, 23-32, 23-33, 23-34, 23-35, 23-36, 24-32, 24-33, 24-34, 24-36, 25-31, 25-32, 25-33, 26-32 | 12-24, 13-25, 11-27, 12-26, 14-24, 15-23, 12-25, 13-27, 11-26, 15-25, 11-28, 13-24, 12-27, 16-22, 14-22, 14-23, 15-22, 23-34, 25-32, 17-23, 15-24, 16-23, 23-32, 21-34, 24-32, 24-33, 14-25, 25-31, 26-32, 8-24, 23-33, 10-28, 26-33, 24-34, 22-34, 22-35, 24-35, 13-26, 10-27 |
| 2YSG | GSSGSSGLPYGWEEAYTADGIKYFINHVTQTTSWIHPVMS:CCCCCCCCCCCEEEEECCCCCEEEEECCCCCEECCCCCCC | 40 | 7-38 | 20 | 5-36, 5-38, 7-38, 8-14, 8-36, 8-37, 8-38, 9-37, 9-38, 11-26, 11-27, 11-28, 11-29, 12-24, 12-25, 12-26, 12-27, 12-28, 12-37, 13-23, 13-24, 13-25, 13-26, 13-27, 14-22, 14-23, 14-24, 14-25, 15-21, 15-22, 15-23, 15-24, 15-25, 16-22, 16-23, 17-23, 22-34, 23-32, 23-33, 23-34, 23-35, 24-32, 24-33, 24-34, 24-35, 24-36, 25-31, 25-32, 25-33, 25-34, 26-32, 26-33 | 12-24, 13-25, 12-26, 11-27, 14-24, 12-25, 15-23, 11-26, 13-27, 15-25, 13-24, 11-28, 16-22, 12-27, 14-22, 14-23, 15-22, 17-23, 23-34, 25-32, 15-24, 16-23, 23-32, 21-34, 24-32, 24-33, 14-25, 25-31, 10-28, 26-32, 8-24, 23-33, 22-35, 26-33, 24-34, 22-34, 24-35, 13-26, 10-27 |
| 2YSH | GSSGSSGLPPGWQSYLSPQGRRYYVNTTTNETTWERPSSS:CCCCCCCCCCCCEEEECCCCCEEEECCCCCCEECCCCCCC | 40 | 7-37 | 20 | 4-34, 4-35, 4-36, 5-35, 5-36, 8-36, 8-37, 9-37, 11-26, 12-24, 12-25, 12-26, 12-27, 12-37, 13-24, 13-25, 13-26, 13-27, 14-22, 14-23, 14-24, 14-25, 15-22, 15-23, 15-24, 15-25, 16-22, 16-23, 16-24, 17-23, 22-34, 22-35, 23-33, 23-34, 23-35, 23-36, 24-32, 24-33, 24-34, 24-35, 24-36, 24-37, 25-31, 25-32, 25-33, 25-37, 26-32, 26-37, 29-40 | 13-27, 11-27, 13-25, 12-24, 11-28, 12-26, 14-24, 12-25, 15-23, 11-26, 14-22, 15-25, 16-22, 12-27, 13-24, 14-23, 15-22, 17-23, 25-32, 23-34, 15-24, 16-23, 23-32, 14-25, 21-34, 24-32, 25-31 |
| 2YSI | GSSGSSGTEEIWVENKTPDGKVYYYNARTRESAWTKPDGV:CCCCCCCCCCCEEEEECCCCCEEEEECCCCCEECCCCCCC | 40 | 9-40 | 20 | 10-36, 10-40, 11-26, 11-27, 11-28, 12-24, 12-25, 12-26, 12-27, 12-36, 13-24, 13-25, 13-26, 13-27, 14-22, 14-23, 14-24, 14-25, 15-22, 15-23, 15-24, 15-25, 16-22, 16-23, 17-23, 22-34, 23-32, 23-33, 23-34, 23-35, 24-32, 24-33, 24-34, 24-35, 24-36, 25-31, 25-32, 25-33, 26-32 | 12-24, 13-25, 12-26, 11-27, 14-24, 15-23, 12-25, 13-27, 15-25, 11-28, 12-27, 13-24, 14-22, 11-26, 16-22, 14-23, 15-22, 25-32, 17-23, 23-34, 15-24, 16-23, 23-32, 24-32, 10-27, 24-33, 26-32, 25-31, 14-25, 24-34, 21-34, 23-33, 26-33, 25-33, 10-28, 22-35, 13-26 |

**Table S5**. Dataset 3: long linear peptides

| PDB | SEQUENCE :  SEC. STRUCTURE | LEN | RIGID CORE | NMR No. | NATIVE CONTACTS | Raptor-X CONTACTS |
| --- | --- | --- | --- | --- | --- | --- |
| 1DV0 | GSQEKEAIERLKALGFPESLVIQAYFACEKNENLAANFLLSQNFDDE:CCCCHHHHCCCCCCCCCHHHHHHHHCCCCCCHHHHHHHCCCCCCCCC | 47 | 4-42 | 18 | 2-28, 5-23, 5-30, 6-16, 6-19, 6-20, 6-23, 7-16, 9-16, 9-19, 9-33, 9-34, 9-37, 10-16, 10-19, 18-37, 19-37, 22-33, 22-36, 22-37, 23-29, 23-30, 23-32, 23-33, 23-36, 26-32, 26-33, 26-36 | 24-38, 24-35, 24-39, 8-21, 11-21, 20-39, 7-32, 25-35, 28-35, 16-39, 11-36, 21-39, 8-22, 8-25, 11-35, 28-34, 10-32, 12-21, 7-25, 27-38, 11-39, 12-18, 14-36, 8-18, 11-32, 28-38, 11-25, 4-25, 14-40, 7-30, 20-42, 27-35, 14-39, 21-35, 11-24, 23-38, 27-34, 20-44, 7-31, 7-35, 24-36, 4-30, 10-36, 25-32 |
| 1F4I | QEKEAIERLKALGFEESLVIQAYFACEKNENLAANFLLSQNFDDE:CCHHHHHHHHHCCCCHHHHHHHHHCCCCCCHHHHHHHHHCCCCCC | 45 | 2-40 | 21 | 2-23, 2-28, 6-16, 6-19, 6-20, 7-16, 9-15, 9-16, 9-19, 9-33, 9-34, 9-37, 10-16, 10-19, 14-37, 18-37, 19-33, 19-37, 22-33, 22-34, 22-36, 22-37, 23-29, 23-30, 23-32, 23-33, 26-32, 26-33, 26-36 | 22-36, 22-33, 22-37, 6-19, 9-19, 18-37, 23-33, 5-30, 26-33, 14-37, 9-34, 19-37, 6-20, 6-23, 9-33, 26-32, 10-19, 8-30, 25-36, 12-34, 10-16, 9-37, 5-23, 6-16, 9-30, 26-36, 9-23, 12-38, 12-37, 25-33, 18-40, 2-23, 19-33, 5-28, 9-22, 21-36, 25-32, 22-34, 8-34, 5-29, 23-30 |
| 1IFY | TLVTGSEYETMLTEIMSMGYERERVVAALRASYNNPHRAVEYLLTGIPG:CCCCCHHHHHHHHHHHHCCCCHHHHHHHHHCCCCCCHHHHHHHHHCCCC | 49 | 5-48 | 10 | 8-26, 8-34, 8-36, 9-22, 9-26, 11-25, 11-26, 11-35, 11-36, 11-37, 11-39, 12-20, 12-21, 12-22, 12-23, 12-25, 12-26, 12-36, 13-21, 13-22, 15-21, 15-25, 15-36, 15-37, 15-39, 15-40, 16-22, 16-25, 20-40, 20-43, 24-43, 25-36, 25-39, 25-40, 25-42, 25-43, 26-34, 26-39, 28-34, 28-38, 28-39, 28-40, 28-42, 28-43, 28-47, 29-35, 29-36, 29-38, 29-39, 31-39, 31-42, 31-47, 32-38, 32-39, 32-42 | 28-42, 28-43, 28-39, 29-39, 11-36, 12-25, 32-39, 15-25, 24-43, 15-40, 25-43, 20-43, 15-39, 12-26, 32-38, 12-29, 14-36, 11-29, 31-42, 18-40, 32-42, 15-43, 16-25, 12-22, 15-36, 16-22, 8-29, 14-40, 11-34, 31-39, 15-29, 11-39, 25-39, 28-40, 18-43, 29-36, 18-44, 20-44, 27-42, 11-35, 8-26 |
| 1K1V | LTDEELVTMSVRELNQHLRGLSKEEIIQLKQRRRTLKNRGY:CCHHHHHHCCHHHHHHHHCCCCHHHHHHHHHHHHHHCCCCC | 41 | 1-41 | 20 | 3-32, 3-33, 3-36, 4-36, 4-41, 6-14, 6-32, 6-33, 6-36, 7-33, 7-36, 7-37, 18-26 | 21-29, 18-26, 6-36, 9-17, 18-29, 6-32, 7-36, 6-33, 6-14, 3-36, 14-29, 6-29, 3-32 |
| 1ND9 | TDVTIKTLAAERQTSVERLVQQFADAGIRKSADDSVSAQEKQTLIDHLN:CEECCCHHHHHHCCCHHHHHHHHHHHCCCCCCCCCEECCHHHHHHHHHC | 49 | 1-42:45-49 | 10 | 1-37, 1-38, 1-39, 2-36, 2-37, 2-38, 3-35, 3-36, 3-37, 3-38, 4-33, 4-34, 4-35, 4-36, 4-37, 5-16, 5-17, 5-20, 5-29, 5-30, 5-31, 5-32, 5-33, 5-34, 5-35, 5-36, 5-37, 6-17, 6-31, 6-32, 6-33, 6-34, 6-36, 7-33, 8-16, 8-17, 8-36, 8-38, 9-15, 9-16, 9-17, 9-18, 9-20, 9-31, 9-36, 10-16, 10-17, 11-17, 15-39, 15-41, 16-36, 16-37, 16-38, 16-39, 16-40, 16-41, 17-31, 17-36, 18-31, 18-41, 18-43, 18-44, 19-31, 19-37, 19-40, 19-41, 19-42, 19-43, 19-44, 20-28, 20-29, 20-30, 20-31, 20-35, 20-36, 20-37, 20-40, 20-41, 20-43, 21-29, 21-30, 21-31, 22-30, 22-43, 22-44, 22-47, 23-29, 23-30, 23-40, 23-43, 23-47, 24-30, 24-31, 26-43, 26-47, 28-36, 28-37, 29-35, 29-36, 29-37, 30-36 | 4-35, 3-36, 2-35, 3-35, 4-34, 5-34, 5-36, 4-33, 2-36, 3-41, 5-32, 2-37, 9-16, 9-19, 6-32, 26-44, 6-33, 5-16, 4-36, 5-19, 8-36, 26-43, 22-47, 28-40, 5-30, 4-32, 5-33, 8-19, 23-44, 6-16, 5-20, 1-38, 22-44, 8-44, 22-48, 26-47, 1-37, 12-48, 5-31, 9-15, 8-45, 5-35, 8-41, 28-44, 2-38, 28-43, 26-40, 19-48, 5-23, 12-19, 3-38, 25-47, 14-48, 12-45, 5-44, 1-41, 3-37, 28-36, 30-36 |
| 1P9C | MTISQQEFGRTGLPDLSSMTEEEQIAYAMQMSLQGAEFGQAESAD:CCCCCCCCCCCCCCCHHHHHHHHHHHHHHHHHCCCCCCCCCCCCC | 45 | 16-33 | 10 | 5-14, 9-15, 16-23, 16-24, 32-38 | 19-27, 16-27, 16-24, 14-27 |
| 1PGY | ALVDEVKDMEIARLMSLGLSIEEATEFYENDVTYERYLEILKSKQKE:CCCHHHHHHHHHHHHHHCCCCHHHHHHHHHHCCCHHHHHHHHHCCCC | 47 | 4-44 | 20 | 7-25, 10-28, 10-33, 10-34, 11-24, 11-25, 11-28, 13-34, 14-24, 14-25, 14-28, 14-34, 17-38, 28-34, 28-37, 31-37 | 14-24, 11-24, 10-28, 14-27, 11-28, 15-24, 14-21, 11-21, 7-28, 19-27, 15-21, 14-37, 11-25, 10-34, 17-41, 10-24, 14-34, 14-28, 27-37, 11-37, 28-34, 6-37, 3-34 |
| 1PV0 | MRKLSDELLIESYFKATEMNLNRDFIELIENEIKRRSLGHIISVSS:CCCCCHHHHHHHHHHHHHHCCCHHHHHHHHHHHHHCCCCCCCCCCC | 46 | 3-44 | 25 | 1-9, 1-29, 1-32, 6-33, 9-29, 9-33, 10-29, 10-33, 10-41, 10-42, 12-26, 12-29, 13-25, 13-26, 13-29, 13-30, 13-42, 14-42, 15-21, 16-22, 16-25, 16-26, 17-26, 30-39, 30-42, 33-39, 39-45, 39-46, 40-46 | 13-29, 9-33, 12-29, 16-26, 9-29, 9-32, 16-29, 10-33, 13-26, 6-36, 16-25, 13-30, 6-38, 13-33, 17-26, 6-33, 9-38, 9-36, 10-38, 4-32, 21-29, 4-36, 10-42, 6-37 |
| 1USE | GSPSSSDYSDLQRVKQELLEEVKKELQKVKEEIIEAFVQELRKRGSP:CCCCCCCHHHHHHHHHHHHHHHHHHHHHHHHHHHHHHHHHHHHCCCC | 47 | 5-44 | 1 |  |  |
| 1VPU | LQIDRLIDRITERAEDSGNESEGDQEELSALVERGHLAPWDVDDL:CCHHHHHHHHHHCCCHHHHCCCCHHHHCCCCCCHHHCCHHHHCCC | 45 | 5-17:23-29:39-42 | 9 | 4-25, 4-26, 4-27, 4-28, 4-29, 4-30, 5-26, 7-25, 7-26, 7-38, 7-39, 8-17, 8-25, 8-26, 8-38, 8-39, 10-39, 11-17, 11-38, 11-39, 11-40, 11-42, 11-43, 12-18, 12-39, 13-43, 14-39, 14-42, 14-43, 14-45, 15-42, 16-42, 17-25, 17-37, 17-38, 17-39, 17-42, 20-37, 21-37, 25-37, 25-38, 29-38, 30-36 | 6-31, 10-28, 6-37, 6-28, 3-31, 3-37, 31-37, 7-28, 10-24, 32-38, 3-32, 10-42, 10-31, 10-39, 7-39, 6-39, 13-20, 6-27, 14-42, 3-39 |
| 1W4E | GSNRRVIAMPSVRKYAREKGVDIRLVQGTGKNGRVLKEDIDAWLAGG:CCCCCCCCCCCHHHHHHHCCCCHHHCCCCCCCCCCCHHHHHHHHHCC | 47 | 6-46 | 20 | 3-22, 4-21, 4-22, 5-21, 5-26, 5-31, 5-32, 5-33, 6-21, 6-26, 6-32, 6-33, 6-34, 7-32, 7-33, 7-34, 9-35, 10-33, 10-34, 10-35, 10-38, 11-20, 11-21, 13-19, 13-35, 13-38, 14-20, 14-21, 14-35, 14-38, 17-38, 17-42, 19-38, 19-41, 19-42, 21-33, 21-38, 23-41, 24-33, 24-37, 24-38, 24-41, 25-31, 25-32, 25-33, 25-37, 26-32, 26-33, 26-34, 26-37, 27-33, 27-34, 27-37, 28-34 | 15-40, 12-40, 12-35, 8-35, 12-37, 19-44, 11-37, 9-35, 16-40, 9-34, 7-34, 15-37, 12-36, 21-44, 7-35, 7-33, 6-35, 8-23, 21-43, 8-34, 9-36, 6-33, 16-23, 25-43, 26-43, 6-23, 6-34, 21-40, 26-39, 26-40, 28-35, 26-35, 9-37, 28-34, 12-23, 7-32, 6-24, 6-26, 15-41, 8-26, 19-40, 5-33, 8-36, 16-22, 6-32, 27-35, 6-27, 27-39, 29-35, 13-23, 5-32, 8-16, 6-28, 11-36, 15-44, 7-23 |
| 1W4G | GSNRRVIAMPSVRKWAREKGVDIRLVQGTGKNGRVLKEDIDAFLAGG:CCCCCCCCCHHHHHHHHHHCCCHHHCCCCCCCCCCCHHHHHHHHCCC | 47 | 6-46 | 20 | 4-21, 4-22, 5-26, 5-31, 5-32, 5-33, 6-21, 6-26, 6-28, 6-31, 6-32, 6-33, 6-34, 7-32, 7-33, 7-34, 10-21, 10-33, 10-34, 10-35, 10-38, 11-20, 11-21, 13-19, 13-35, 13-38, 14-20, 14-21, 14-38, 19-38, 21-33, 21-38, 24-33, 24-37, 24-38, 24-41, 25-31, 25-32, 25-33, 25-37, 26-32, 26-33, 26-34, 26-37, 26-38, 27-33, 27-34, 27-37, 28-34 | 12-40, 12-35, 15-40, 8-35, 12-37, 19-44, 9-35, 7-34, 16-40, 9-34, 11-37, 12-36, 15-37, 21-44, 7-35, 7-33, 21-43, 6-35, 8-23, 8-34, 9-36, 6-33, 26-43, 16-23, 25-43, 6-23, 6-34, 21-40, 26-39, 26-40, 28-35, 26-35, 9-37, 12-23, 28-34, 7-32, 6-24, 19-40, 6-26, 8-26, 5-33, 8-36, 6-32, 16-22, 6-27, 15-41, 27-35, 27-39, 13-23, 29-35, 5-32, 6-28, 11-36, 7-23, 8-16, 21-47 |
| 1W4H | GSQNNDALSPAIRRLLAEHNLDASAIKGTGVGGRLTREDVEKHLAKA:CCCCCCCCCHHHHHHHHHCCCCHHHCCCCCCCCCCCHHHHHHHHHHC | 47 | 7-46 | 20 | 2-30, 3-30, 3-32, 4-32, 5-26, 5-31, 5-32, 5-33, 6-21, 6-32, 6-33, 7-33, 7-34, 9-35, 10-21, 10-33, 10-34, 10-35, 10-38, 11-21, 13-38, 14-20, 14-21, 14-38, 21-33, 21-38, 24-33, 24-37, 24-38, 24-41, 25-31, 25-32, 25-33, 25-37, 26-32, 26-33, 26-34, 26-37, 27-33, 27-34, 27-37, 28-34 | 15-40, 12-40, 12-35, 12-37, 19-44, 11-37, 8-35, 15-37, 16-40, 9-34, 9-35, 26-43, 21-44, 21-43, 8-23, 7-34, 12-36, 25-43, 26-39, 16-23, 8-34, 26-40, 9-36, 7-35, 28-35, 26-35, 7-33, 15-41, 28-34, 21-40, 9-37, 21-47, 6-33, 12-23, 15-44, 8-26, 6-23, 29-35, 27-35, 16-22, 6-34, 27-39, 7-32, 8-16, 28-39, 8-36, 11-36, 9-33, 7-28, 29-36 |
| 1WR7 | GSPGIQSFLPPGWEMRIAPNGRPFFIDHNTKTTTWEDPRLK:CCCCCCCCCCCCEEEEECCCCCEEEEECCCCEEECCCHHHC | 41 | 9-40 | 20 | 5-11, 9-38, 9-39, 12-27, 12-28, 12-29, 13-25, 13-26, 13-27, 13-28, 13-29, 14-25, 14-26, 14-27, 14-28, 15-23, 15-24, 15-25, 15-26, 16-23, 16-24, 16-25, 16-26, 17-23, 17-24, 18-24, 22-35, 23-35, 24-33, 24-34, 24-35, 24-36, 24-37, 25-33, 25-34, 25-35, 25-36, 25-37, 26-32, 26-33, 26-34, 27-33 | 13-25, 14-26, 13-27, 12-28, 15-25, 13-26, 16-24, 12-27, 14-28, 16-26, 17-23, 13-28, 14-25, 12-29, 15-23, 15-24, 16-23, 18-24, 24-35, 26-33, 17-24, 16-25, 24-33, 9-25, 15-26, 22-35, 25-33, 25-34, 26-32, 27-33, 24-34, 25-35, 11-29, 23-35, 14-27, 27-34 |
| 1YMZ | GSHGRSMPLPPGWERRTDVEGKVYYFNVRTLTTTWERPTIILE:CCCCCCCCCCCCEEEEECCCCCEEEEECCCCEEECCCCCCCCC | 43 | 9-19:21-38 | 10 | 1-32, 1-33, 1-34, 1-35, 2-33, 2-34, 2-35, 3-33, 3-34, 3-35, 4-33, 4-34, 4-35, 4-36, 6-22, 6-23, 6-24, 7-20, 7-21, 7-22, 7-23, 7-24, 7-33, 8-20, 8-21, 8-22, 8-23, 9-18, 9-19, 9-20, 9-21, 10-18, 10-19, 10-20, 10-21, 11-17, 11-18, 11-19, 12-18, 12-19, 18-30, 19-28, 19-29, 19-30, 19-31, 20-28, 20-29, 20-30, 20-31, 20-32, 20-33, 21-27, 21-28, 21-29, 22-28, 22-29 | 13-25, 12-28, 13-27, 14-26, 15-25, 13-26, 16-24, 14-28, 12-27, 13-28, 16-26, 14-25, 17-23, 15-23, 12-29, 15-24, 16-23, 24-35, 26-33, 18-24, 16-25, 24-33, 17-24, 9-25, 25-33, 25-34, 22-35, 26-32, 27-33, 15-26, 25-35, 27-34, 24-34, 23-36, 25-36, 26-34, 10-25, 23-35, 23-38, 14-27 |
| 1YWJ | GSRRASVGSAKSMWTEHKSPDGRTYYYNTETKQSTWEKPDD:CCCCCCCCCCCCCEEEEEEHHHEEEEEECCCCEEECCCCCC | 41 | 12-39 | 15 | 2-17, 2-18, 2-19, 3-15, 3-16, 3-17, 3-18, 3-28, 4-15, 4-16, 4-17, 4-18, 5-13, 5-14, 5-15, 5-16, 6-12, 6-13, 6-14, 6-15, 7-13, 7-14, 8-14, 13-25, 14-23, 14-24, 14-25, 14-26, 15-22, 15-23, 15-24, 15-25, 15-26, 15-28, 16-22, 16-23, 16-24, 17-23 | 14-26, 15-27, 14-28, 13-29, 17-25, 16-26, 14-27, 15-29, 17-27, 13-28, 13-30, 15-26, 14-29, 16-24, 18-24, 16-25, 17-24, 19-25, 27-34, 25-36, 17-26, 25-34, 18-25, 26-34, 12-29, 28-34, 27-33, 26-36, 26-35, 16-27, 23-36, 28-35, 25-35, 27-35, 17-34 |
| 2BTG | GSQNNDALSPAIRRLLAEHNLDASAIKGTGVGGRLTREDVEKWLAKA:CCCCCCCCCHHHHHHHHHHCCCHHHCCCCCCCCCCCHHHHHHHHHCC | 47 | 7-46 | 20 | 4-32, 5-26, 5-31, 5-32, 5-33, 6-26, 6-32, 6-33, 6-34, 7-33, 7-34, 10-34, 10-35, 14-20, 14-21, 14-38, 24-37, 24-38, 24-41, 25-31, 25-33, 25-37, 26-32, 26-33, 26-34, 26-37, 27-33, 27-34, 27-37, 28-34 | 15-40, 12-40, 12-35, 12-37, 19-44, 11-37, 8-35, 15-37, 9-34, 16-40, 9-35, 21-44, 26-43, 7-34, 8-23, 21-43, 12-36, 25-43, 8-34, 16-23, 7-35, 9-36, 26-40, 26-39, 28-35, 7-33, 26-35, 15-41, 28-34, 9-37, 21-47, 21-40, 12-23, 6-33, 15-44, 8-26, 16-22, 27-35, 6-23, 6-34, 29-35, 7-32, 8-16, 27-39, 8-36, 7-28, 11-36, 28-39, 9-33, 29-36, 12-26, 15-21 |
| 2DMV | GSSGSSGLPPGWEQRVDQHGRVYYVDHVEKRTTWDRPSGPSSG:CCCCCCCCCCCEEEEECCCCCEEEEECCCCCEECCCCCCCCCC | 43 | 7-37 | 20 | 8-14, 11-26, 11-27, 11-28, 12-24, 12-25, 12-26, 12-27, 13-24, 13-25, 13-26, 13-27, 14-22, 14-23, 14-24, 14-25, 15-22, 15-23, 15-24, 15-25, 16-22, 16-23, 17-23, 21-34, 22-34, 22-35, 23-32, 23-33, 23-34, 23-35, 24-32, 24-33, 24-34, 24-35, 24-36, 25-31, 25-32, 25-33, 26-32 | 12-24, 13-25, 12-26, 11-27, 14-24, 15-23, 12-25, 11-26, 13-27, 15-25, 16-22, 14-22, 11-28, 13-24, 12-27, 14-23, 15-22, 17-23, 23-34, 25-32, 23-32, 15-24, 24-33, 24-32, 16-23, 25-31, 21-34, 14-25, 26-32, 8-24, 23-33, 24-34, 26-33, 22-34, 22-35, 10-28, 25-33, 13-26 |
| 2E5T | IDVLRAKAAKERAERRLQSQQDDIDFKRAELALKRAMNRLSVAEMK:CCHHHHHHHHHHHHHHHCCCCCCCCHHHHHHHHHHHHHHHHHHHCC | 46 | 2-19:21-46 | 20 | 1-39, 1-40, 1-43, 6-36, 10-33, 10-36, 13-29, 13-33, 16-25, 17-25, 17-26, 17-29 | 6-40, 13-33, 9-36, 6-39, 6-36, 10-36, 17-29, 13-32, 13-29, 16-29, 3-43, 10-33, 3-40, 9-32, 13-36, 17-26, 7-40, 10-37, 1-39, 20-26, 1-43, 10-40, 14-33, 9-35, 6-43, 2-43, 12-32, 17-30, 16-25, 5-39, 17-33, 20-29, 3-44, 9-33, 16-32, 2-39, 9-39, 16-28, 6-37, 3-39, 1-40, 13-30, 7-36, 1-42, 14-29 |
| 2EKK | GSSGSSGVNQQQLQQLMDMGFTREHAMEALLNTSTMEQATEYLLTHP:CCCCCCCCCHHHHHHHHHHHCCHHHHHHHHHHCCCHHHHHHHHHCCC | 47 | 8-46 | 20 | 8-27, 8-30, 8-31, 9-30, 12-30, 12-36, 12-39, 13-23, 13-26, 13-27, 13-30, 16-26, 16-39, 16-40, 16-43, 17-23, 17-26, 21-43, 21-47, 25-43, 25-46, 26-39, 26-43, 29-39, 29-42, 29-43, 30-36, 30-38, 30-39, 33-39, 33-42 | 29-42, 29-43, 29-39, 13-26, 30-39, 16-26, 33-39, 13-27, 12-36, 26-43, 25-43, 21-43, 13-30, 8-30, 12-30, 17-23, 16-40, 33-42, 17-26, 13-23, 8-27, 16-39, 32-42, 8-31, 16-43, 15-36, 16-30, 25-46, 6-31, 16-36, 19-40, 7-31, 29-46, 32-39, 19-44, 19-43, 30-36, 26-39, 16-29, 28-42, 29-40 |
| 2J8P | HMTPQDHEKAALIMQVLQLTADQIAMLPPEQRQSILILKEQIQKSTGAP:CCCHHHHCHHHHHHHHHCCCHHHHHCCCHHHHCHHHHHHHHHHCCCCCC | 49 | 2-44 | 30 | 6-33, 7-29, 7-32, 7-33, 8-32, 8-33, 9-32, 9-33, 9-35, 9-36, 10-32, 10-33, 10-34, 10-35, 10-36, 11-21, 11-24, 13-35, 13-36, 13-39, 14-20, 14-21, 14-24, 15-21, 24-32, 40-46 | 24-35, 24-32, 16-35, 27-35, 16-38, 17-39, 13-42, 13-38, 17-42, 24-36, 16-39, 19-35, 17-38, 19-39, 25-32, 16-42, 17-35, 10-42, 16-24, 12-38 |
| 2JNH | EAALENVDAKIAKLMGEGYAFEEVKRALEIAQNNVEVARSILREFA:CCCCCCHHHHHHHHHCCCCCHHHHHHHHHHCCCCHHHHHHHHCCCC | 46 | 6-43 | 15 | 8-26, 8-33, 9-22, 9-23, 9-26, 11-33, 12-22, 12-33, 12-37, 13-22, 17-40, 21-40, 22-36, 22-40, 25-33, 25-35, 25-36, 25-39, 25-40, 26-32, 26-33, 26-36, 29-35, 29-36 | 27-41, 27-42, 14-24, 11-24, 27-38, 31-38, 28-38, 11-28, 24-42, 30-41, 15-24, 31-37, 10-35, 15-21, 11-25, 14-38, 19-42, 23-42, 10-28, 14-39, 31-41, 14-42, 14-35, 7-28, 13-35, 11-21, 23-45, 30-38, 17-39, 14-28, 27-45, 14-27, 30-37, 17-42, 26-41, 28-35, 24-38 |
| 2K9D | VIRSIIKSSRLEEDRKRYLMTLLDDIKGANDLAKFHQMLVKIIM:CHHHHHHHHCCCCHHHHHHHHHHHCCCCCHHHHHHHHHHHHHHC | 44 | 1-9:11-26:28-43 | 20 | 1-25, 3-24, 3-33, 3-34, 3-37, 4-21, 4-24, 4-25, 6-37, 7-17, 7-20, 7-21, 7-37, 8-17, 8-18, 8-21, 10-17, 24-33, 27-33 | 27-36, 23-39, 6-20, 27-39, 7-20, 3-24, 12-20, 24-36, 3-20, 3-40, 6-40, 20-43, 23-43, 20-40, 23-40, 3-36, 23-36, 27-33, 3-23, 7-43, 19-43, 16-43, 3-43, 12-44, 24-40, 27-35 |
| 2L4J | GSPNSSPASGPLPEGWEQAITPEGEIYYINHKNKTTSWLDPRLETR:CCCCCCCCCCCCCCCCEEEECCCCCEEEEECCCCEEECCCCCCCCC | 46 | 12-39 | 21 | 1-7, 9-43, 10-42, 10-43, 11-43, 12-18, 12-42, 13-42, 14-31, 15-29, 15-30, 15-31, 15-32, 16-28, 16-29, 16-30, 16-31, 16-32, 16-42, 17-28, 17-29, 17-30, 17-31, 18-26, 18-27, 18-28, 18-29, 19-26, 19-27, 19-28, 19-29, 20-26, 20-27, 20-28, 21-27, 27-36, 27-37, 27-38, 28-36, 28-37, 28-38, 28-39, 28-41, 29-35, 29-36, 29-37, 30-36, 30-37 | 16-28, 17-29, 16-30, 15-31, 18-28, 19-27, 16-29, 17-31, 15-30, 19-29, 20-26, 16-31, 17-28, 18-26, 15-32, 18-27, 19-26, 27-38, 29-36, 21-27, 27-36, 28-37, 28-36, 19-28, 20-27, 29-35, 25-38, 12-28, 27-37, 30-36, 18-29, 28-38, 30-37, 28-39, 26-39, 26-38, 13-28, 29-37, 12-41, 27-39, 17-30, 26-41 |
| 2P81 | AKREFNENRYLTERRRQQLSSELGLNEAQIKIWFQNKRAKIKKS:CCCCCCCCCCCHHHHHHHHHHHHCCCHHHHHHHHHHHHHHCCCC | 44 | 8-38 | 25 | 16-27, 16-30, 19-25, 20-26, 20-27, 20-30 | 20-30, 19-30, 20-27, 16-30, 16-27, 20-26, 11-34, 17-27, 16-31, 5-37, 23-30, 10-34, 5-41, 16-34, 19-25, 10-38, 5-38, 25-33, 20-29, 9-41, 5-34, 2-37, 11-19, 11-31, 6-41 |
| 2WXC | GSQNNDALSPAIRRLLAEWNLDASAIKGTGVGGRLTREDVEKHLAKA:CCCCCCCCCHHHHHHHHHHCCCHHHCCCCCCCCCCCHHHHHHHHHHC | 47 | 7-47 | 20 | 6-34, 7-23, 7-33, 7-34, 7-35, 8-23, 8-34, 8-35, 8-36, 9-35, 9-36, 11-37, 12-35, 12-36, 12-37, 12-40, 15-21, 16-22, 16-23, 23-35, 26-35, 26-39, 26-40, 26-43, 27-34, 27-35, 27-39, 27-43, 28-34, 28-35, 28-36, 28-39, 29-35, 29-36, 29-39, 30-36 | 12-40, 15-40, 12-35, 12-37, 11-37, 19-44, 8-35, 15-37, 16-40, 9-34, 9-35, 26-43, 21-44, 7-34, 12-36, 21-43, 8-23, 25-43, 8-34, 9-36, 7-35, 16-23, 26-40, 26-39, 28-35, 7-33, 26-35, 15-41, 28-34, 21-40, 9-37, 21-47, 6-33, 8-26, 12-23, 6-34, 15-44, 27-35, 16-22, 29-35, 7-32, 27-39, 6-23, 8-36, 8-16, 7-28, 11-36, 28-39, 6-35, 29-36, 6-32, 25-47, 15-21 |
| 2YSB | GSSGSSGEDLPLPPGWSVDWTMRGRKYYIDHNTNTTHWSHPLESGPSSG:CCCCCCCCCCCCCCCEEEEECCCCCEEEEECCCCEEECCCCCCCCCCCC | 49 | 9-43 | 20 | 7-39, 7-40, 10-40, 10-42, 12-40, 12-41, 12-42, 15-30, 15-31, 15-32, 16-28, 16-29, 16-30, 16-31, 16-32, 17-28, 17-29, 17-30, 17-31, 18-26, 18-27, 18-28, 18-29, 19-26, 19-27, 19-28, 19-29, 20-26, 20-27, 21-27, 26-38, 27-36, 27-37, 27-38, 27-39, 28-36, 28-37, 28-38, 28-39, 28-40, 28-41, 29-35, 29-36, 29-37, 30-36, 42-48 | 16-28, 16-30, 15-31, 17-29, 18-28, 16-29, 19-27, 17-31, 15-30, 16-31, 20-26, 15-32, 18-26, 17-28, 19-29, 18-27, 19-26, 27-38, 29-36, 21-27, 27-36, 28-37, 12-28, 28-36, 20-27, 19-28, 25-38, 29-35, 27-37, 30-36, 28-38, 18-29, 30-37, 12-41, 10-40, 26-38, 29-37, 11-40, 26-39, 12-40, 13-28, 28-39, 10-42 |
| 2ZAJ | GSSGSSGLDSELELPAGWEKIEDPVYGIYYVDHINRKTQYENPSGPSSG:CCCCCCCCCCCCCCCCCEEEEEECCCEEEEEECCCCEEECCCCCCCCCC | 49 | 9-44 | 20 | 6-43, 7-41, 7-42, 7-43, 7-44, 7-45, 8-45, 9-42, 9-44, 9-45, 10-42, 10-44, 12-44, 12-45, 13-44, 14-42, 14-43, 14-44, 15-44, 17-31, 17-32, 17-33, 17-34, 17-35, 18-30, 18-31, 18-32, 18-33, 18-34, 19-30, 19-31, 19-32, 20-28, 20-29, 20-30, 20-31, 21-27, 21-28, 21-29, 21-30, 21-31, 22-28, 22-29, 28-40, 29-38, 29-39, 29-40, 30-38, 30-39, 30-40, 30-41, 31-37, 31-38, 31-39, 32-38 | 18-30, 19-31, 18-32, 17-33, 21-29, 20-30, 18-31, 21-31, 19-33, 17-32, 22-28, 20-28, 19-30, 17-34, 18-33, 20-29, 21-28, 31-38, 23-29, 29-40, 14-30, 30-39, 29-38, 21-30, 30-38, 31-37, 22-29, 32-38, 32-39, 30-40, 29-39, 20-31, 30-41, 28-41, 27-40, 28-40, 28-43, 31-39, 14-43, 15-30, 12-42, 29-41 |
| 3E21 | GSMDREMILADFQACTGIENIDEAITLLEQNNWDLVAAINGVIPQ:CCCCHHHHHHHHHHHHCCCCHHHHHHHHHHCCCCHHHHHCCCCCC | 45 | 3-42 | 1 | 3-31, 6-31, 6-33, 7-22, 7-23, 7-26, 9-33, 10-16, 10-22, 10-33, 10-36, 10-37, 11-17, 11-22, 13-37, 14-37, 14-40, 16-22, 25-36, 26-32, 26-33, 26-36, 28-36, 29-35, 29-36 | 9-24, 12-24, 9-25, 13-24, 9-28, 28-38, 8-35, 27-38, 9-21, 8-28, 27-42, 12-35, 13-21, 15-39, 12-39, 31-38, 24-42, 12-28, 27-41, 16-42, 5-28, 28-35, 13-19, 12-38, 18-24, 23-42, 16-39, 31-37, 18-42, 8-33, 12-27, 16-43, 16-24, 13-20, 11-35, 24-38, 31-41, 5-33, 15-42, 15-43, 10-21, 9-35, 5-25, 18-43 |

**Table S6**. Dataset 4: cyclic peptides closed by disulfide bridge

| PDB | SEQUENCE :  SEC. STRUCTURE | LEN | RIGID CORE | NMR No. | NATIVE CONTACTS | Raptor-X CONTACTS |
| --- | --- | --- | --- | --- | --- | --- |
| 1B45 | GRCCHPACGKYYSC:CCCCCHHCCCCCCC | 14 | 2-9,13-14 | 43 | 1-11, 1-12, 1-13, 2-11, 2-12, 2-13, 3-11, 3-12, 3-13, 3-14, 4-12, 4-13, 4-14, 5-12, 5-14, 6-12 |  |
| 1ETL | CELCCNPGCAGC:CCCCCCCCCCCC | 12 | - | 1 | 1-9, 1-10, 1-12, 4-12, 6-12 |  |
| 1GNB | TCEICAYAACTGC:CCCCCCCCCCCCC | 13 | 2-7 | 20 | 1-8, 1-9, 1-10, 2-8, 2-9, 2-10, 3-10, 5-13, 6-13, 7-13 |  |
| 1HJE | ICCNPACGPKYSC:CCCHHHHHHHCCC | 13 | - | 1 | 1-10, 1-11, 1-12, 1-13, 2-10, 2-11, 2-12, 2-13, 3-11, 3-12, 3-13, 4-11, 4-12, 5-11 |  |
| 1HP9 | GHACYRNCWREGNDEETCKERC:CCCCCHHHHHHHCCCHHHHHHC | 22 | - | 1 | 4-18, 4-21, 4-22, 5-15, 5-18, 5-19, 5-22, 8-18, 9-15, 9-17, 9-18 |  |
| 1IEN | FNWRCCLIPACRRNHKKFC:CCHHHHHHHHHHCCHHHHC | 19 | 2-19 | 20 | 3-18, 3-19, 5-11, 5-12, 5-15, 6-12, 6-15, 6-16, 6-18, 6-19, 12-18 |  |
| 1IM1 | GCCSDPRCAWRC:CHHCCHHCCCCC | 12 | - | 20 | 2-12, 2-8, 2-9, 3-10, 3-12, 3-9 |  |
| 1IM7 | IWGCSGKLICTTA:CCCCCCCCCCCCC | 13 | - | 1 | 2-10, 3-10, 4-10 |  |
| 1JBL | GRCTKSIPPICFPD:CCCCCCCCCCCCCC | 14 | - | 20 | 1-11, 1-12, 1-13, 1-14, 2-11, 2-12, 2-13, 2-14, 3-10, 3-11, 3-12, 3-9, 4-10, 4-11 |  |
| 1KCN | ALCPAVCYVGGKALCPDVCYV:CCCHHHHCCCCCCCCHHHHHC | 21 | 3-9,14-21 | 20 | 2-19, 3-15, 3-19, 6-12, 6-13, 6-14, 7-13, 7-14, 7-15, 8-20 |  |
| 1KWD | PCSICSNNPTCWAICK:CCCCCCCCCCCCCCCC | 16 | 2-12,14-15 | 16 | 1-15, 1-16, 2-12, 2-15, 2-16, 3-12, 3-15, 5-11, 5-12 |  |
| 1MII | GCCSNPVCHLEHSNLC:CCCCCCHHHHCCCCCC | 16 | - | 20 | 2-12, 2-8, 2-9, 3-12, 3-13, 3-15, 3-16, 3-9, 4-16, 9-15, 9-16 |  |
| 1MMC | VGECVRGRCPSGMCCSQFGYCGKGPKYCGR:CCCCCCCCCCCCCECCCCCCECCCHHHCCC | 30 | 3-29 | 26 | 1-20, 1-21, 1-22, 2-20, 2-21, 3-19, 3-20, 3-21, 4-15, 4-19, 4-20, 4-21, 6-19, 7-15, 7-19, 8-14, 8-15, 9-15, 9-21, 12-23, 13-21, 13-22, 13-23, 13-24, 14-21, 14-22, 14-23, 14-24, 14-27, 14-28, 15-21, 15-22, 15-27, 15-28, 16-22, 16-26, 16-27, 16-28, 16-29, 17-27, 17-28, 17-29, 17-30, 18-27, 20-27, 21-27, 21-28, 22-28 |  |
| 1N0A | CTWEPDGKLTC:CECCCCCCECC | 11 | - | 21 | 1-10, 1-11, 1-9, 2-10, 2-11, 2-9, 3-10, 3-9, 4-10 |  |
| 1N0C | CHWEGNKLVC:CEECCCEECC | 10 | - | 20 | 1-9, 1-10, 1-8, 2-9, 2-10, 2-8, 3-9 |  |
| 1NIM | KCTSDQDEQFIPKGCSK:CCCCCCCCCCCCCCCCC | 17 | 5,7-8,10-14 | 25 | 1-14, 1-15, 1-16, 1-17, 2-11, 2-12, 2-14, 2-15, 2-16, 3-11, 3-15 |  |
| 1OIG | RPECVLNSDCPSNQACVNQKCRDP:CCCCCCCCCCCCCCECCCCCECCC | 24 | 2-11,14-23 | 22 | 2-19, 2-20, 3-19, 3-20, 3-21, 4-10, 4-16, 4-17, 4-19, 4-20, 4-21, 5-16, 5-19, 5-20, 5-21, 6-15, 6-16, 7-13, 7-14, 7-15, 7-16, 7-21, 8-14, 8-15, 9-16, 10-16, 10-21, 14-21, 14-22, 14-23, 14-24, 15-21, 15-22, 15-23, 15-24, 16-22, 16-23, 16-24, 17-23 |  |
| 1ORX | VCGETCVGGTCNTPGCTCSWPVCT:CCCCCCCCCCCCCCCCCCCCCCCC | 24 | - | 20 | 1-23, 1-24, 2-13, 2-16, 2-23, 2-24, 3-22, 3-23, 4-11, 4-12, 4-13, 4-22, 4-23, 5-11, 5-20, 5-21, 5-22, 5-23, 6-18, 6-19, 6-20, 6-21, 6-22, 6-23, 7-20, 7-21, 7-22, 8-20, 9-18, 9-20, 10-18, 11-17, 11-18, 11-23, 13-23, 16-23, 16-24, 17-23, 17-24, 18-24 |  |
| 1R8T | RCCHPQCGAAYSCRK:CCCCCCHHHHCCCCC | 15 | 1-14 | 30 | 1-11, 1-12, 1-13, 2-11, 2-12, 2-13, 3-11, 3-12, 3-13, 4-11 |  |
| 1RPC | CLGIGSCNDFAGCGYAVVCFW:CCCCCCCCCCCCCCCCCCCCC | 21 | 1-11,13-19 | 20 | 1-10, 1-13, 1-14, 1-15, 1-16, 1-8, 1-9, 2-14, 2-15, 2-16, 2-9, 3-14, 3-15, 3-16, 4-15, 4-16, 4-17, 5-15, 5-16, 5-17, 6-15, 6-16, 6-17, 7-14, 7-15, 7-16, 7-17, 7-19, 8-14, 8-15, 8-16, 8-19, 8-20, 9-15, 9-16, 9-18, 9-19, 9-20 |  |
| 1SP7 | PCPPVCVAQCVPTCPQYCCPAKRK:CCHHHHHHCCCCCCCCCCCCCCCC | 24 | - | 10 | 2-14, 2-15, 2-18, 3-14, 5-11, 6-14, 6-15, 6-18, 6-19, 7-18, 7-19, 7-20, 9-19, 10-19, 10-20 |  |
| 1TER | ALCNCNRIIIPHMCWKKCGKK:CCCCCCCCCCCHHHHHHHCCC | 21 | 2-21 | 21 | 2-14, 3-10, 3-11, 3-14, 4-10, 4-11, 4-14, 5-14, 5-15, 5-18, 8-21, 9-21, 10-21, 11-21, 12-21, 14-20, 14-21, 15-21 |  |
| 1V5A | RCLPSGKACAGVTQKIPCCGSCVRGKCS:CCCCCCCCCCCCCCCCCCCCCECCCECC | 28 | 1-9,12,14,15,17-28 | 15 | 1-15, 1-16, 1-19, 2-16, 2-17, 2-18, 2-19, 2-20, 3-16, 3-17, 3-18, 3-19, 3-20, 3-27, 3-9, 4-18, 4-19, 4-20, 4-27, 4-28, 5-18, 5-19, 5-20, 5-22, 5-26, 5-27, 5-28, 6-26, 6-27, 6-28, 7-26, 7-27, 8-25, 8-26, 8-27, 9-17, 9-18, 9-22, 9-23, 9-25, 9-26, 9-27, 10-25, 10-26, 18-27, 18-28, 19-27, 20-27, 20-28, 21-27, 21-28, 22-28 | 21-27, 21-28, 22-28, 3-9, 20-27 |
| 1V6R | CSCSSLMDKECVYFCHLDIIW:CCCCCCCCHHHHHCCCCCCCC | 21 | - | 20 | 1-14, 1-15, 1-16, 2-14, 2-15, 3-11, 3-12, 3-14, 3-15, 4-11, 5-11, 5-12, 6-12, 6-15, 10-19, 12-19, 13-19, 13-20 |  |
| 1WM8 | VGCEECPMHCKGKNANPTCDDGVCNCNV:CCCCCCHHHHCCCCCCCCCCCCCCCCCC | 28 | - | 10 | 17-24, 18-25, 17-26, 18-24, 17-25, 15-26, 16-26, 19-25, 15-27, 16-27, 18-27, 18-26, 15-28 | 17-24, 18-25, 17-26, 18-24, 17-25, 15-26, 16-26, 19-25, 15-27, 16-27, 18-27, 18-26, 15-28 |
| 1WQC | DPCYEVCLQQHGNVKECEEACKHPVE:HHHHHHHHHHCCCHHHHHHHHHCCCC | 26 | - | 30 | 3-18, 3-21, 4-14, 4-17, 4-18, 4-21, 7-13, 7-17, 8-14 | 7-17, 8-14, 8-17 |
| 1X7K | RRWCFRVCYRGRFCYRKCR:CCCCCCECCCCCCECCCCC | 19 | 8-9,14,16-18 | 16 | 3-18, 4-16, 4-17, 4-18, 4-19, 5-16, 5-17, 5-18, 6-14, 6-15, 6-16, 6-17, 6-18, 7-14, 7-15, 7-16, 8-14, 8-15 |  |
| 1XGB | ECCNPACGRHYSC:CCCCCCCCCCCCC | 13 | 2-13 | 24 | 1-11, 1-12, 1-13, 2-11, 2-12, 2-13, 3-10, 3-11, 3-12, 3-13, 3-9, 4-11 |  |
| 2AJW | GCCSNPVCHLEHSNLCGGAAGG:HHHCHHHHHHHCCHHHHCCCCC | 22 | 1-2,5-13,17 | 20 | 1-11, 1-12, 1-13, 2-11, 2-12, 2-13, 3-10, 3-11, 3-12, 3-13, 3-9, 4-11 |  |
| 2EFZ | VCCPFGGCHELCYCCD:CCCCCCCCCCCCCCCC | 16 | - | 20 | 2-12, 2-14, 2-15, 2-16, 3-12, 3-14, 3-15, 3-16, 4-15, 4-16, 5-15, 5-16, 8-15, 8-16, 9-15 |  |
| 2I28 | GCCSTPPCAVLYC:CCCCHHHHHCCCC | 13 | - | 20 | 2-13, 2-8, 2-9, 3-12, 3-13, 3-9, 4-13, 5-13 |  |
| 2IT7 | GCPRILMRCKQDSDCLAGCVCGPNGFCG:CCCCCCEECCHHHHCCCCCEECCCEEEC | 28 | - | 30 | 1-17, 1-18, 1-19, 2-16, 2-17, 2-18, 2-19, 2-28, 3-16, 3-17, 3-19, 3-28, 4-28, 6-26, 6-27, 6-28, 7-15, 7-26, 7-27, 8-14, 8-15, 8-25, 8-26, 8-27, 9-15, 9-21, 9-25, 9-26, 9-27, 10-21, 10-25, 10-26, 11-20, 11-21, 12-19, 12-20, 12-21, 12-27, 14-21, 15-21, 15-27, 15-28, 16-27, 19-27, 19-28, 20-27, 20-28, 21-27, 21-28, 22-28 | 21-27, 19-27, 9-27, 20-27, 6-26, 9-21, 9-25, 15-27, 9-26, 19-28, 8-25 |
| 2NX7 | AQNPCSLQQPGCSSACAPACRLSCCSLG:CCCHHHHHCCCCHHHHCCCCHHHHHCCC | 28 | 2-28 | 10 | 2-14, 3-13, 3-14, 3-17, 4-14, 5-12, 5-13, 5-14, 5-17, 5-20, 5-25, 6-12, 6-13, 6-14, 6-25, 9-25, 9-26, 10-25, 10-26, 11-25, 11-26, 12-20, 12-24, 12-25, 12-26, 12-27, 12-28, 13-20, 13-24, 13-25, 13-26, 13-28, 14-20, 16-24, 16-25, 16-28 |  |
| 2OQ9 | APMQAPVQAAPACMASCAPQCCGR:CCCCCCCCCCCCCCCCCCCCCCCC | 24 | 8-22 | 10 | 10-22, 11-21, 11-22, 11-23, 12-21, 12-22, 13-19, 13-20, 13-21, 13-22, 13-23, 14-21, 14-22 |  |

**Table S7**. Dataset 5: cyclic peptides closed by backbone

| PDB | SEQUENCE :  SEC. STRUCTURE | LEN | RIGID CORE | NMR No. | NATIVE CONTACTS | Raptor-X CONTACTS |
| --- | --- | --- | --- | --- | --- | --- |
| 1BH4 | CGESCVWIPCISAALGCSCKNKVCYRNGIP:CCCCCCCCCCCCCCCCCCCCCCCCCCCCCC | 30 | 1:10,12:13,16:30 | 12 | 1-13, 1-17, 1-24, 1-25, 1-26, 1-29, 1-30, 2-13, 2-23, 2-24, 2-25, 2-30, 3-22, 3-23, 3-24, 3-25, 3-30, 4-19, 4-21, 4-22, 4-23, 4-24, 5-19, 5-22, 5-23, 5-24, 6-22, 6-23, 9-19, 9-24, 10-19, 10-24, 13-19, 13-24, 13-25, 13-26, 14-24, 16-26, 17-24, 17-25, 17-26, 17-27, 18-24, 18-25, 18-26, 18-27, 19-25, 19-26, 20-27, 24-30 | 17-24, 18-25, 18-24, 17-26, 17-25, 16-26, 18-27, 19-25, 16-27, 17-27, 18-26 |
| 1HVZ | GFCRCLCRRGVCRCICTR:CCCCEEECCCEEECCCCC | 18 | 1:7,12:12,14:14,16:17 | 20 | 1-17, 1-18, 2-16, 2-17, 2-18, 3-14, 3-15, 3-16, 3-17, 3-18, 4-14, 4-15, 4-16, 5-12, 5-13, 5-14, 5-15, 6-12, 6-13, 6-14, 6-15, 7-13, 7-14 |  |
| 1IB9 | SGSDGGVCPKILKKCRRDSDCPGACICRGNGYCG:CCCCCCCCCCCCCCCCHHHHCCCCCECCCCCCEC | 34 | 7:9,11:34 | 20 | 1-25, 1-26, 1-27, 1-33, 1-34, 1-7, 1-8, 1-9, 2-25, 2-26, 2-34, 2-8, 5-24, 5-25, 6-24, 6-25, 6-26, 6-34, 7-23, 7-24, 7-25, 7-26, 7-34, 8-23, 8-24, 8-25, 8-26, 8-33, 8-34, 11-32, 11-33, 11-34, 12-32, 12-33, 13-21, 13-32, 13-33, 14-20, 14-21, 14-31, 14-32, 14-33, 15-21, 15-27, 15-28, 15-31, 15-32, 15-33, 16-27, 16-31, 16-32, 17-26, 17-27, 18-25, 18-26, 18-27, 18-28, 18-33, 18-34, 19-25, 19-26, 19-27, 20-26, 20-27, 20-33, 21-27, 21-32, 21-33, 21-34, 25-33, 25-34, 26-33, 26-34, 27-33, 27-34, 28-34 | 15-33, 25-33, 27-33, 15-27, 12-32, 26-33, 15-31, 15-32, 13-32, 12-33, 14-31, 14-32, 13-33, 26-34, 25-34, 21-33, 12-34, 27-34 |
| 1JJZ | NGLPVCGETCVGGTCNTPGCTCSWPVCTR:CCCCCCCCCCCCCCCCCCCCCCCCCCCCC | 29 | 01:29 | 20 | 1-20, 1-21, 1-28, 1-29, 2-21, 2-23, 2-28, 2-29, 3-28, 3-29, 4-26, 4-27, 4-28, 4-29, 5-27, 5-28, 5-29, 6-17, 6-20, 6-26, 6-27, 6-28, 6-29, 7-26, 7-27, 7-28, 8-15, 8-16, 8-17, 8-26, 8-27, 9-15, 9-22, 9-24, 9-25, 9-26, 9-27, 10-16, 10-22, 10-23, 10-24, 10-25, 10-26, 10-27, 11-24, 11-25, 11-26, 13-22, 13-24, 14-22, 15-21, 15-22, 15-26, 15-27, 19-29, 20-27, 20-28, 20-29, 21-27, 21-28, 21-29, 22-28 | 20-27, 21-27, 20-28, 21-28, 10-27 |
| 1N1U | AGETCVGGTCNTPGATCSWPVCTRNGLPV:CCCCCCCCCCCCCCCEECCCEEECCCECC | 29 | 01:29 | 20 | 1-28, 1-29, 2-21, 2-28, 2-29, 3-10, 3-11, 3-12, 3-21, 3-22, 3-23, 3-28, 3-29, 4-10, 4-19, 4-20, 4-21, 4-22, 5-17, 5-18, 5-19, 5-20, 5-21, 5-22, 6-19, 6-20, 6-21, 8-17, 8-19, 9-17, 10-16, 10-17, 10-22, 12-29, 14-24, 15-22, 15-23, 15-24, 15-29, 16-22, 16-23, 16-24, 17-23, 21-28, 22-28, 22-29, 23-29 | 15-22, 16-22, 16-23, 15-24, 15-23, 10-22, 16-25, 14-24, 5-22, 17-23, 15-25, 5-17 |
| 1NBJ | CAESCVYIPCTVTALLGCSCSNRVCYNGIP:CCCCCCCCCCHHHHHCCCEECCCEECCCCC | 30 | 01:30 | 20 | 1-13, 1-18, 1-24, 1-25, 1-26, 1-27, 1-29, 1-30, 2-13, 2-25, 2-30, 3-10, 3-11, 3-12, 3-13, 3-24, 3-25, 4-10, 4-20, 4-23, 4-24, 4-25, 5-20, 5-21, 5-23, 5-24, 5-25, 6-23, 6-24, 7-23, 9-20, 10-18, 10-19, 10-20, 10-25, 13-25, 16-27, 17-27, 18-25, 18-26, 18-27, 19-25, 19-26, 19-27, 20-26 | 18-25, 19-25, 19-26, 18-26, 18-27, 17-27, 19-28, 18-28, 17-28, 20-26, 19-27 |
| 1PT4 | CGETCFGGTCNTPGCSCTWPICTRDGLPV:CCCCCCCCCCCCCCCEECCCEECCCCCCC | 29 | 01:29 | 20 | 1-12, 1-15, 1-22, 1-23, 1-24, 1-27, 1-28, 1-29, 2-21, 2-22, 2-28, 2-29, 3-10, 3-11, 3-12, 3-21, 3-22, 4-10, 4-17, 4-19, 4-20, 4-21, 4-22, 5-17, 5-18, 5-19, 5-20, 5-21, 5-22, 6-19, 6-20, 6-21, 7-20, 8-17, 8-19, 8-20, 9-17, 10-17, 10-22, 14-24, 15-22, 15-23, 15-24, 15-25, 16-22, 16-23, 16-24, 16-25, 16-26, 17-23, 18-26, 21-28, 22-28, 23-29 | 15-22, 16-22, 16-23, 15-23, 15-24, 10-22, 16-25, 14-24, 17-23, 5-22, 14-25, 15-25, 5-17 |
| 1PXQ | NKGCATCSIGAACLVDGPIPDFEIAGAGLFGLWG:CCCCCCCCCCCCCCCCCCCCCCCCCCCCCCCCCC | 34 | 4:9,11:15,19:32 | 8 | 1-31, 1-32, 1-33, 1-34, 1-35, 2-32, 2-35, 3-31, 3-32, 3-35, 4-29, 4-30, 4-31, 4-32, 5-29, 5-31, 5-32, 6-26, 7-26, 7-27, 7-29, 8-26, 8-29, 8-31, 8-32, 9-26, 9-27, 9-29, 10-24, 10-26, 10-27, 10-29, 10-30, 11-24, 11-26, 11-27, 11-29, 11-30, 12-22, 12-23, 12-24, 13-20, 13-21, 13-22, 13-23, 13-24, 14-20, 14-21, 14-22, 14-23, 14-24, 15-21, 15-22, 15-23 |  |
| 1R1F | TFCGETCRVIPVCTYSAALGCTCDDRSDGLCKRNGDP:CCCCCCCCCCCCCCHHHHHCCCCCCCCCCCCCCCCCC | 37 | 1:26,28:37 | 20 | 1-31, 1-32, 1-33, 1-34, 1-36, 1-37, 2-30, 2-31, 2-32, 2-33, 2-37, 3-16, 3-21, 3-31, 3-32, 3-33, 3-34, 3-37, 4-16, 4-31, 4-32, 4-33, 5-13, 5-15, 5-16, 5-30, 5-31, 6-13, 6-23, 6-28, 6-29, 6-30, 6-31, 7-13, 7-23, 7-25, 7-27, 7-28, 7-29, 7-30, 7-31, 8-25, 8-27, 8-28, 8-29, 9-27, 9-28, 11-23, 11-25, 11-28, 12-22, 12-23, 12-31, 13-21, 13-22, 13-23, 13-31, 16-33, 19-33, 19-34, 20-33, 20-34, 21-31, 21-32, 21-33, 21-34, 21-37, 22-31, 22-32, 22-33, 22-34, 22-37, 23-29, 23-30, 23-31, 23-32, 24-30, 24-31, 24-32, 25-31 | 23-31, 21-31, 24-32, 21-33, 22-31, 21-32, 22-32, 24-31 |
| 1T9E | GRATKSIPPIAFPD:CCCCCCCCCCCCCC | 14 | 01:14 | 20 | 1-11, 1-12, 1-13, 1-14, 2-10, 2-11, 2-12, 2-13, 2-14, 3-10, 3-11, 3-12, 3-9, 4-10, 4-11 |  |
| 1VB8 | CAESCVWIPCTVTALLGCSCSNKVCYNGIP:CCCCCCCCCCCCHHHHCCCCCCCCCCCCCC | 30 | 1:6,8:30 | 20 | 1-13, 1-18, 1-25, 1-26, 1-27, 1-29, 1-30, 2-13, 2-25, 2-30, 3-10, 3-12, 3-13, 3-24, 3-25, 4-10, 4-20, 4-23, 4-24, 4-25, 5-20, 5-22, 5-23, 5-24, 5-25, 6-23, 6-24, 7-23, 9-20, 10-18, 10-19, 10-20, 10-24, 10-25, 13-25, 14-25, 17-27, 18-25, 18-26, 18-27, 18-28, 18-29, 19-25, 19-26, 19-27, 20-26, 21-27 | 18-25, 19-25, 19-26, 18-26, 18-27, 17-27, 19-28, 18-28, 17-28, 20-26, 19-27 |
| 1YP8 | CGESCFLGTCYTKGCSCGEWKLCYGTNGGTIFD:CCCCCCCCCCCCCCEEECCCCEEEEECCCEECC | 33 | 01:33 | 20 | 1-12, 1-15, 1-22, 1-23, 1-24, 1-25, 1-32, 1-33, 2-22, 2-23, 2-33, 3-10, 3-11, 3-12, 3-15, 3-22, 3-23, 4-10, 4-11, 4-22, 4-23, 5-11, 5-17, 5-21, 5-22, 5-23, 6-21, 8-17, 8-21, 9-16, 9-17, 10-16, 10-17, 10-23, 12-25, 13-25, 14-24, 14-25, 14-26, 14-27, 15-23, 15-24, 15-25, 15-26, 15-27, 15-33, 16-22, 16-23, 16-24, 16-25, 16-26, 17-23, 17-24, 18-24, 22-33, 23-33, 24-31, 24-32, 24-33, 25-31, 25-32, 25-33, 26-32 | 15-23, 17-23, 16-23, 15-24, 15-25, 16-24 |
| 1ZA8 | CGESCAMISFCFTEVIGCSCKNKVCYLNSIS:CCECCCCCCCHHHHCCCCEECCCEECCCCCC | 31 | 01:31 | 20 | 1-13, 1-18, 1-25, 1-26, 1-27, 1-30, 1-31, 2-25, 2-26, 2-31, 3-11, 3-12, 3-13, 3-24, 3-25, 3-26, 3-31, 4-11, 4-20, 4-23, 4-24, 4-25, 5-11, 5-20, 5-21, 5-23, 5-24, 5-25, 6-23, 6-24, 9-20, 9-23, 10-19, 10-20, 11-18, 11-19, 11-20, 11-25, 13-19, 13-25, 14-20, 14-25, 17-27, 18-25, 18-26, 18-27, 18-28, 19-25, 19-26, 19-27, 20-26, 24-31, 25-31 | 18-25, 19-26, 19-25, 18-26, 18-27, 19-28, 17-27, 20-26, 18-28, 17-28, 5-25 |
| 2B38 | CGETCLLGTCYTTGCTCNKYRVCTKDGSVLN:CCCCCCCCCCCCCCCEECCCCEECCCCCCCC | 31 | 01:25 | 20 | 1-12, 1-15, 1-23, 1-24, 1-25, 1-28, 1-29, 1-30, 1-31, 2-22, 2-23, 2-24, 2-28, 2-29, 2-30, 2-31, 3-10, 3-22, 3-23, 3-24, 4-10, 4-17, 4-21, 4-22, 4-23, 5-17, 5-21, 5-22, 5-23, 6-21, 9-16, 9-17, 10-16, 10-17, 10-23, 14-25, 15-23, 15-24, 15-25, 16-22, 16-23, 16-24, 16-25, 17-23, 17-24, 18-24, 23-29, 24-31, 25-31 | 15-23, 17-23, 16-23, 15-25, 15-24, 16-24, 18-24, 10-23, 16-26, 14-25, 5-17, 16-25, 5-23, 15-26, 14-26 |
| 2BEY | CTKSIPPICTKSIPPI:CCCCCCCCCCCCCCCC | 16 | 01:16 | 20 | 1-10, 1-15, 1-16, 1-7, 1-8, 1-9, 2-16, 2-8, 2-9, 9-15, 9-16, 10-16 |  |
| 2ERI | CGESCVFIPCISTLLGCSCKNKVCYRNGVIP:CCECCCCCCCCCCCCCCEECCCEECCCCCCC | 31 | 01:31 | 20 | 1-17, 1-24, 1-25, 1-26, 1-30, 1-31, 2-23, 2-24, 2-25, 2-31, 3-10, 3-11, 3-17, 3-23, 3-24, 3-25, 3-31, 4-10, 4-19, 4-22, 4-23, 4-24, 5-19, 5-20, 5-22, 5-23, 5-24, 6-22, 6-23, 9-19, 10-17, 10-18, 10-19, 10-24, 11-17, 15-26, 16-25, 16-26, 16-27, 17-24, 17-25, 17-26, 17-27, 18-24, 18-25, 18-26, 18-28, 19-25, 23-31, 24-31, 25-31 | 17-24, 18-24, 17-26, 18-25, 17-25, 16-26, 19-25, 18-27, 10-24, 5-24, 17-27, 16-27 |
| 2F2I | CGETCVGGTCNTPGCTCSWDKCTRNGLPV:CCECCCCCCCCCCCCEECCCEECCCCCCC | 29 | 01:29 | 20 | 1-12, 1-15, 1-22, 1-23, 1-24, 1-28, 1-29, 2-21, 2-22, 2-23, 2-28, 2-29, 3-10, 3-11, 3-21, 3-22, 3-23, 4-10, 4-17, 4-20, 4-21, 4-22, 5-17, 5-20, 5-21, 5-22, 6-20, 7-20, 8-16, 8-17, 8-18, 8-20, 9-17, 10-16, 10-17, 10-22, 14-24, 15-22, 15-23, 15-24, 16-22, 16-23, 16-24, 17-23, 21-28, 22-28, 22-29, 23-29 | 15-22, 16-22, 16-23, 15-24, 15-23, 16-25, 14-24, 10-22, 17-23, 5-22, 14-25, 15-25, 16-24, 5-17 |
| 2F2J | CGETCVGGTCNTPGCTCSKNKCTRNGLPV:CCCCCCCCCCCCCCEEECCCEEECCCCCC | 29 | 01:29 | 20 | 1-12, 1-15, 1-21, 1-22, 1-23, 1-24, 1-27, 1-28, 1-29, 2-21, 2-22, 2-23, 2-28, 2-29, 3-10, 3-11, 3-12, 3-21, 3-22, 4-10, 4-17, 4-20, 4-21, 4-22, 5-17, 5-18, 5-20, 5-21, 5-22, 8-17, 8-18, 9-16, 9-17, 10-16, 10-17, 10-22, 12-22, 14-24, 14-25, 15-22, 15-23, 15-24, 15-25, 16-22, 16-23, 16-24, 16-25, 16-26, 17-23, 21-28, 22-28, 22-29, 23-29 | 15-22, 16-23, 16-22, 15-24, 15-23, 16-25, 14-24, 5-22, 17-23, 10-22, 14-25, 15-25, 16-24, 5-17 |
| 2GJ0 | GSIPACGESCFKGKCYTPGCSCSKYPLCAKN:CHHHHHCCCCCCCCCCCCCEEEECCCEEECC | 31 | 1:11,14:31 | 20 | 1-20, 1-21, 1-23, 1-29, 1-30, 1-31, 2-29, 2-30, 2-31, 3-24, 3-27, 3-28, 3-29, 3-30, 4-29, 5-29, 5-30, 6-17, 6-20, 6-28, 6-29, 6-30, 7-27, 7-28, 7-29, 8-15, 8-16, 8-17, 8-20, 8-27, 8-28, 8-29, 9-15, 9-22, 9-25, 9-26, 9-27, 9-28, 10-22, 10-25, 10-26, 10-27, 10-28, 11-26, 11-27, 14-22, 15-21, 15-22, 15-28, 19-30, 19-31, 20-28, 20-29, 20-30, 20-31, 21-28, 21-29, 21-30, 21-31, 22-28, 22-29, 23-29 | 20-28, 22-28, 23-29, 21-28, 20-30, 20-29, 21-29, 21-31, 15-28, 19-30, 10-28, 20-31, 10-22, 22-29, 19-31 |
| 2K7G | GVPICGETCTLGTCYTAGCSCSWPVCTRN:CEEEEEECCHHCCCCCCCEEECCCEEECC | 29 | 01:29 | 20 | 1-20, 1-27, 1-28, 1-29, 2-27, 2-28, 2-29, 3-25, 3-26, 3-27, 3-28, 4-27, 4-28, 5-16, 5-19, 5-26, 5-27, 5-28, 6-25, 6-26, 6-27, 7-14, 7-15, 7-25, 7-26, 7-27, 8-14, 8-23, 8-24, 8-25, 8-26, 9-21, 9-22, 9-23, 9-24, 9-25, 9-26, 10-23, 10-24, 10-25, 11-24, 12-21, 12-23, 12-24, 13-21, 13-26, 14-20, 14-21, 14-26, 18-28, 18-29, 19-26, 19-27, 19-28, 19-29, 20-26, 20-27, 20-28, 20-29, 21-27 | 19-26, 20-26, 20-27, 19-27, 19-28, 14-26, 21-27, 20-29 |
| 2KNM | GIPCGESCVWIPCISSAIGCSCKSKVCYRN:CEEEEECCCCCCHHHHHHCCEECCCEEECC | 30 | 01:30 | 20 | 1-28, 1-29, 1-30, 2-28, 2-29, 2-30, 3-26, 3-27, 3-28, 3-29, 4-15, 4-20, 4-26, 4-27, 4-28, 4-29, 5-26, 5-27, 5-28, 6-12, 6-13, 6-14, 6-15, 6-26, 6-27, 7-13, 7-22, 7-25, 7-26, 7-27, 8-22, 8-25, 8-26, 8-27, 9-25, 9-26, 13-20, 13-21, 13-22, 13-27, 14-20, 15-21, 15-27, 16-27, 18-29, 19-29, 20-27, 20-28, 20-29, 20-30, 21-27, 21-28, 21-29, 22-28 | 20-27, 21-27, 20-28, 20-29, 21-28, 8-27, 19-29, 22-28, 21-30, 20-30, 19-30, 8-22 |
| 2KUK | CGETCFTGTCYTNGCTCDPWPVCTRNGLPV:CCCCCCCCCCCCCCCCCCCCCCCCCCCCCC | 30 | 01:30 | 20 | 1-15, 1-22, 1-23, 1-24, 1-25, 1-28, 1-29, 1-30, 2-22, 2-23, 2-29, 2-30, 3-10, 3-11, 3-12, 3-22, 3-23, 4-10, 4-11, 4-17, 4-20, 4-21, 4-22, 4-23, 5-11, 5-17, 5-20, 5-21, 5-22, 5-23, 6-20, 6-21, 6-22, 7-21, 8-17, 8-20, 8-21, 9-16, 9-17, 10-16, 10-17, 10-23, 14-24, 14-25, 14-26, 15-23, 15-24, 15-25, 15-26, 15-27, 16-23, 16-24, 16-25, 17-23, 17-24, 18-24, 19-29, 22-29, 23-29, 23-30, 24-30 | 15-23, 17-23, 16-23, 15-25, 16-24, 15-24, 18-24, 10-23, 16-26, 5-23, 5-17, 14-25 |
| 2KUX | GTPCGESCVYIPCISGVIGCSCTDKVCYLN:CEEEEECCCCCCCHHHHHCCEECCCEEECC | 30 | 01:30 | 20 | 1-28, 1-29, 1-30, 2-28, 2-29, 2-30, 3-20, 3-26, 3-27, 3-28, 3-29, 4-15, 4-16, 4-20, 4-27, 4-28, 4-29, 5-26, 5-27, 5-28, 6-13, 6-15, 6-16, 6-26, 6-27, 6-28, 7-13, 7-22, 7-25, 7-26, 7-27, 8-22, 8-25, 8-26, 8-27, 9-25, 9-26, 13-22, 13-27, 16-22, 16-27, 16-28, 16-29, 18-29, 19-29, 19-30, 20-27, 20-28, 20-29, 20-30, 21-27, 21-28, 21-29, 21-30, 22-28 | 20-27, 21-27, 20-28, 20-29, 21-28, 8-27, 19-29, 21-30, 22-28, 20-30, 19-30, 8-22 |
| 2KVX | GSLCGDTCFVLGCNDSSCSCNYPICVKD:CEEEEECCCCCCCCCCCECCCCCEEECC | 28 | 1-30 | 20 | 1-26, 1-27, 1-28, 2-26, 2-27, 2-28, 3-24, 3-25, 3-26, 3-27, 4-15, 4-18, 4-24, 4-25, 4-26, 4-27, 5-24, 5-25, 5-26, 6-13, 6-14, 6-24, 6-25, 7-13, 7-20, 7-22, 7-23, 7-24, 7-25, 8-20, 8-21, 8-22, 8-23, 8-24, 8-25, 9-20, 9-22, 9-23, 9-24, 12-20, 13-19, 13-20, 13-25, 17-26, 17-27, 17-28, 18-25, 18-26, 18-27, 18-28, 19-25, 19-26, 19-27, 20-26 | 18-25, 19-25, 19-26, 18-26, 18-27, 8-25, 20-26 |
| 2LAM | GLPTCGETCTLGTCYVPDCSCSWPICMKN:CCCCCCCCCCCCCCCCCCCEECCCEECCC | 29 | 01:29 | 20 | 1-22, 1-27, 1-28, 1-29, 2-27, 2-28, 2-29, 3-25, 3-26, 3-27, 3-28, 4-26, 4-27, 4-28, 5-16, 5-19, 5-26, 5-27, 5-28, 6-25, 6-26, 7-14, 7-16, 7-25, 7-26, 8-14, 8-21, 8-23, 8-24, 8-25, 8-26, 9-21, 9-22, 9-23, 9-24, 9-25, 9-26, 10-23, 10-24, 10-25, 12-21, 12-23, 12-24, 13-21, 14-20, 14-21, 14-26, 18-28, 19-26, 19-27, 19-28, 20-26, 20-27, 20-28, 20-29, 21-27 | 19-26, 20-26, 20-27, 19-27, 19-28, 20-29, 14-26, 21-27, 18-28, 9-26 |
| 2LUR | CGETCVGGTCNTPGCTCSWPVCGHFRWGV:CCCCCCCCCCCCCCCEECCCEECCCCCCC | 29 | 01:29 | 20 | 1-15, 1-22, 1-23, 1-24, 1-28, 1-29, 2-22, 2-23, 2-28, 2-29, 3-21, 3-22, 3-23, 3-29, 4-10, 4-17, 4-18, 4-19, 4-20, 4-21, 4-22, 4-23, 5-17, 5-18, 5-19, 5-20, 5-21, 5-22, 6-19, 6-20, 6-21, 7-19, 7-20, 8-17, 8-18, 8-19, 8-20, 9-17, 10-16, 10-17, 10-22, 10-23, 11-17, 15-23, 15-24, 16-22, 16-23, 16-24, 17-23, 17-24, 18-24, 18-25, 18-28, 22-28, 22-29, 23-29 | 15-22 |
| 2M9O | GLPVCGETCTLGTCYTQGCTCSWPICKRN:CCCCCCCCCCCCCCCCCCEEECCCEEECC | 29 | 01:29 | 20 | 1-27, 1-28, 1-29, 2-27, 2-28, 2-29, 3-19, 3-25, 3-26, 3-27, 3-28, 4-26, 4-27, 4-28, 5-16, 5-17, 5-19, 5-26, 5-27, 5-28, 6-25, 6-26, 7-14, 7-15, 7-16, 7-25, 7-26, 8-14, 8-23, 8-24, 8-25, 8-26, 9-21, 9-22, 9-23, 9-24, 9-25, 9-26, 10-23, 10-24, 10-25, 12-21, 12-23, 13-21, 14-20, 14-21, 14-26, 16-26, 17-28, 18-27, 18-28, 18-29, 19-26, 19-27, 19-28, 19-29, 20-26, 20-27, 20-28, 21-27 | 19-26, 20-26, 20-27, 19-27, 19-28, 14-26, 21-27, 20-29, 18-28 |
| 5WOW | GGVCPKILQRCRRDSDCPGACICRGNGYCGYPYDVPDYA:CCCCCCCCCCCCCCCCCCCCCCCCCCCCCCCCCCCCCCC | 39 | 3:30,32:32 | 20 | 1-38, 1-39, 2-20, 2-21, 2-22, 2-30, 2-31, 2-39, 3-20, 3-21, 3-30, 3-31, 3-32, 3-33, 3-38, 3-39, 4-19, 4-20, 4-21, 4-30, 4-31, 4-32, 4-33, 5-32, 5-33, 6-32, 6-33, 7-32, 7-33, 8-28, 8-29, 8-31, 8-32, 9-17, 9-28, 9-29, 10-17, 10-23, 10-27, 10-28, 10-29, 11-17, 11-22, 11-23, 11-24, 11-27, 11-28, 11-29, 12-23, 12-27, 12-28, 13-22, 13-23, 14-21, 14-22, 14-23, 14-29, 15-22, 16-22, 16-23, 17-23, 17-29, 21-29, 21-30, 22-29, 22-30, 23-29, 23-30, 24-30, 31-38 | 23-29, 21-30, 21-29, 22-30, 22-29, 21-31, 11-29, 11-23, 11-27, 11-28, 17-29, 22-31, 23-30, 8-28, 10-27, 24-30, 10-28, 20-32, 17-23, 9-28, 22-32 |

SECTION 2 - COMPARISON ON ALL ATOM RESULTS (AA)

The Tables from S8 to S12 contain the following information:

- PDB - This is a pdb code.
- LEN - This is the number of residues.
- Name of the method i.e.CABS-flex de novo.
- Indication whether this is PRIME or BEST model.
- The number is black is RMSD calculated on full structure and the number in gray is RMSD calculated on rigid core (if applicable).

**Table S8**. Dataset 1: short linear peptides

|  |  | **APPTEST** | | | | **PEP-FOLD 3.5** | | | | **CABS-flex  de novo** | | | | **Alpha Fold** | | **ESM Fold** | |
| --- | --- | --- | --- | --- | --- | --- | --- | --- | --- | --- | --- | --- | --- | --- | --- | --- | --- |
| PDB | LEN | PRIME | | BEST | | PRIME | | BEST | | PRIME | | BEST | | PRIME | | PRIME | |
| 1A13 | 14 | 2.26 | 2.05 | 2.02 | 1.89 | 2.05 | 1.85 | 1.82 | 1.73 | 2.69 | 2.61 | 1.27 | 1.24 | 1.93 | 1.93 | 2.18 | 2.07 |
| 1B03 | 18 | 5.05 | - | 2.85 | - | 2.69 | - | 2.33 | - | 2.50 | - | 1.06 | - | 2.74 | - | 2.33 | - |
| 1C98 | 10 | 2.84 | - | 2.30 | - | 5.39 | - | 2.60 | - | 2.42 | - | 0.67 | - | 1.61 | - | 3.37 | - |
| 1D6X | 13 | 2.53 | 2.50 | 2.34 | 2.16 | 2.94 | 2.94 | 2.29 | 2.20 | 1.92 | 1.96 | 1.11 | 0.81 | 3.58 | 2.72 | 1.58 | 1.36 |
| 1D7N | 14 | 1.08 | 0.69 | 0.89 | 0.61 | 1.04 | 0.64 | 0.97 | 0.62 | 0.95 | 0.52 | 0.67 | 0.30 | 0.74 | 0.45 | 0.78 | 0.49 |
| 1D9J | 20 | 2.06 | 0.89 | 1.05 | 0.61 | 4.38 | 1.18 | 2.58 | 0.98 | 3.39 | 2.36 | 2.46 | 0.59 | 1.71 | 0.83 | 1.99 | 0.85 |
| 1D9L | 17 | 1.47 | 0.47 | 1.25 | 0.39 | 1.71 | 0.67 | 1.67 | 0.62 | 1.76 | 0.55 | 1.11 | 0.30 | 1.45 | 0.34 | 1.37 | 0.48 |
| 1D9M | 18 | 1.95 | 1.31 | 1.66 | 0.58 | 2.62 | 1.16 | 2.27 | 0.97 | 3.18 | 1.48 | 2.28 | 0.47 | 1.93 | 0.53 | 2.74 | 1.16 |
| 1D9O | 20 | 3.17 | 0.44 | 2.47 | 0.34 | 3.10 | 0.51 | 2.78 | 0.43 | 3.30 | 0.47 | 2.04 | 0.30 | 2.80 | 0.42 | 2.58 | 0.48 |
| 1D9P | 20 | 1.88 | 0.54 | 1.62 | 0.46 | 2.18 | 0.63 | 1.92 | 0.57 | 2.96 | 0.41 | 1.91 | 0.29 | 1.75 | 0.37 | 1.90 | 0.41 |
| 1DN3 | 15 | 1.10 | - | 1.02 | - | 1.14 | - | 0.91 | - | 1.17 | - | 0.55 | - | 1.37 | - | 1.72 | - |
| 1DU1 | 20 | 4.94 | - | 4.16 | - | 5.12 | - | 4.88 | - | 3.92 | - | 2.18 | - | 4.93 | - | 5.37 | - |
| 1E0Q | 17 | 3.77 | - | 2.79 | - | 2.31 | - | 0.98 | - | 1.38 | - | 0.86 | - | 1.99 | - | 1.37 | - |
| 1EGS | 9 | 6.52 | - | 3.65 | - | 2.04 | - | 1.05 | - | 2.45 | - | 1.09 | - | 4.37 | - | 6.57 | - |
| 1G89 | 13 | 2.55 | 2.34 | 1.82 | 1.75 | 4.51 | 4.36 | 3.28 | 3.15 | 2.89 | 2.47 | 1.46 | 1.37 | 3.42 | 3.34 | 3.60 | 3.69 |
| 1GJF | 14 | 1.86 | 0.78 | 0.96 | 0.55 | 2.38 | 0.73 | 1.39 | 0.66 | 1.95 | 0.52 | 1.00 | 0.32 | 1.46 | 0.50 | 1.77 | 1.02 |
| 1HU5 | 18 | 1.96 | 1.79 | 1.61 | 1.48 | 1.60 | 1.54 | 1.39 | 1.33 | 1.71 | 1.57 | 1.05 | 0.98 | 1.61 | 1.52 | 1.51 | 1.47 |
| 1HU6 | 18 | 3.26 | 3.25 | 2.51 | 2.50 | 3.43 | 3.43 | 2.71 | 2.43 | 3.16 | 3.00 | 1.44 | 1.27 | 3.31 | 3.22 | 3.61 | 3.53 |
| 1HU7 | 18 | 2.05 | 1.94 | 1.77 | 1.69 | 1.79 | 1.63 | 1.79 | 1.58 | 2.40 | 2.24 | 1.08 | 1.02 | 2.00 | 1.63 | 2.06 | 1.60 |
| 1ID6 | 15 | 5.54 | 5.59 | 4.96 | 5.02 | 5.50 | 5.55 | 4.50 | 4.48 | 3.51 | 3.43 | 2.11 | 2.13 | 5.73 | 5.85 | 5.79 | 5.86 |
| 1IN3 | 12 | 1.24 | - | 1.20 | - | 1.67 | - | 1.55 | - | 1.38 | - | 0.97 | - | 1.15 | - | 1.10 | - |
| 1JAV | 19 | 1.45 | - | 1.43 | - | 6.13 | - | 3.28 | - | 1.41 | - | 1.20 | - | 1.65 | - | 1.69 | - |
| 1KZV | 18 | 1.20 | 1.02 | 0.79 | 0.72 | 1.78 | 1.65 | 1.57 | 1.44 | 1.76 | 1.52 | 0.99 | 0.87 | 1.38 | 1.31 | 1.80 | 1.70 |
| 1L2Y | 20 | 1.59 | - | 1.26 | - | 2.90 | - | 1.18 | - | 2.54 | - | 1.24 | - | 0.44 | - | 0.50 | - |
| 1L3Q | 12 | 5.98 | - | 3.80 | - | 3.92 | - | 2.57 | - | 3.03 | - | 1.85 | - | 7.65 | - | 7.86 | - |
| 1LCX | 13 | 2.25 | 1.20 | 1.80 | 1.12 | 2.51 | 1.46 | 2.42 | 1.38 | 1.99 | 1.37 | 0.94 | 0.76 | 1.85 | 0.81 | 1.72 | 0.88 |
| 1M02 | 12 | 4.23 | 3.13 | 2.39 | 1.86 | 4.30 | 3.33 | 3.01 | 2.75 | 2.50 | 2.13 | 1.36 | 0.97 | 5.55 | 3.75 | 4.57 | 3.51 |
| 1MYU | 12 | 1.73 | - | 1.22 | - | 3.00 | - | 0.96 | - | 1.04 | - | 0.50 | - | 3.17 | - | 4.35 | - |
| 1NIZ | 14 | 2.32 | - | 1.74 | - | 1.68 | - | 1.22 | - | 0.96 | - | 0.63 | - | 1.97 | - | 2.86 | - |
| 1NKF | 16 | 5.41 | - | 4.13 | - | 4.71 | - | 1.56 | - | 2.15 | - | 1.45 | - | 5.75 | - | 1.65 | - |
| 1ODP | 20 | 1.83 | - | 1.34 | - | 1.89 | - | 1.75 | - | 1.66 | - | 0.84 | - | 1.22 | - | 1.76 | - |
| 1P0J | 19 | 1.58 | 1.30 | 1.27 | 1.11 | 1.72 | 1.57 | 1.57 | 1.39 | 1.43 | 1.21 | 0.68 | 0.54 | 1.69 | 1.48 | 1.93 | 1.72 |
| 1P0L | 19 | 1.66 | 1.28 | 1.27 | 1.15 | 1.88 | 1.59 | 1.69 | 1.49 | 1.26 | 1.07 | 0.75 | 0.47 | 1.91 | 1.50 | 1.70 | 1.46 |
| 1P0O | 19 | 1.54 | 1.34 | 1.29 | 1.06 | 1.98 | 1.87 | 1.65 | 1.52 | 1.51 | 1.36 | 0.72 | 0.55 | 1.61 | 1.42 | 2.05 | 1.89 |
| 1P5K | 19 | 1.29 | 1.03 | 1.21 | 0.72 | 1.77 | 1.54 | 1.62 | 1.35 | 1.40 | 1.24 | 1.00 | 0.57 | 1.64 | 1.26 | 1.58 | 1.28 |
| 1PEF | 18 | 0.60 | - | 0.60 | - | 0.90 | - | 0.69 | - | 0.91 | - | 0.37 | - | 0.50 | - | 0.48 | - |
| 1QCM | 11 | 2.95 | 2.99 | 2.72 | 2.69 | 2.49 | 1.90 | 1.10 | 1.02 | 1.01 | 0.65 | 0.70 | 0.51 | 0.98 | 0.87 | 1.05 | 1.04 |
| 1QFA | 13 | 1.01 | - | 0.90 | - | 0.72 | - | 0.66 | - | 0.90 | - | 0.66 | - | 1.12 | - | 1.35 | - |
| 1RPV | 17 | 0.86 | 0.35 | 0.63 | 0.28 | 0.74 | 0.54 | 0.73 | 0.38 | 0.89 | 0.32 | 0.44 | 0.24 | 0.74 | 0.33 | 0.50 | 0.28 |
| 1SOL | 20 | 4.14 | - | 2.56 | - | 3.27 | - | 2.59 | - | 3.88 | - | 1.87 | - | 4.29 | - | 8.22 | - |
| 2BP4 | 16 | 2.17 | 1.64 | 1.64 | 1.60 | 5.45 | 5.21 | 4.12 | 4.06 | 0.65 | 0.54 | 0.39 | 0.27 | 4.27 | 4.21 | 6.53 | 6.32 |
| 2BTA | 15 | 4.53 | 2.61 | 3.08 | 1.70 | 4.50 | 2.60 | 4.36 | 2.51 | 3.90 | 1.98 | 2.25 | 0.43 | 4.37 | 2.47 | 4.49 | 2.53 |
| AVG | | **2.60** | **1.70** | **1.95** | **1.36** | **2.81** | **2.00** | **2.05** | **1.64** | **2.09** | **1.48** | **1.17** | **0.70** | **2.51** | **1.72** | **2.71** | **1.88** |

**Table S9**. Dataset 2: medium linear peptides

|  |  | **APPTEST** | | | | **PEP-FOLD 3.5** | | | | **CABS-flex  *de novo*** | | | | **CABS-flex *contacts*** | | | | **Alpha Fold** | | **ESM Fold** | |
| --- | --- | --- | --- | --- | --- | --- | --- | --- | --- | --- | --- | --- | --- | --- | --- | --- | --- | --- | --- | --- | --- |
| PDB | LEN | PRIME | | BEST | | PRIME | | BEST | | PRIME | | BEST | | PRIME | | BEST | | PRIME | | PRIME | |
| 1BHI | 38 | 4.02 | 1.74 | 3.19 | 1.40 | 7.26 | 4.78 | 4.44 | 2.83 | 6.28 | 4.41 | 3.88 | 2.81 | 4.59 | 2.10 | 2.40 | 1.07 | 2.33 | 0.46 | 2.75 | 0.71 |
| 1BWX | 39 | 7.06 | 0.90 | 5.24 | 0.74 | 4.31 | 1.45 | 4.06 | 1.42 | 10.06 | 2.24 | 7.77 | 0.63 | 8.22 | 1.50 | 5.75 | 0.61 | 4.59 | 1.76 | 4.61 | 1.79 |
| 1BY0 | 27 | 1.68 | 0.98 | 1.42 | 0.96 | 1.65 | 1.38 | 1.37 | 1.22 | 2.14 | 1.74 | 1.12 | 0.76 | *no contacts predicted* | | | | 1.59 | 1.12 | 1.53 | 1.29 |
| 1E0L | 40 | 2.87 | 1.83 | 2.71 | 1.83 | 5.13 | 3.78 | 3.61 | 2.41 | 8.38 | 5.82 | 5.13 | 4.82 | 7.38 | 7.44 | 6.38 | 6.43 | 7.32 | 7.32 | 8.52 | 7.47 |
| 1E0M | 37 | 2.75 | 2.32 | 2.07 | 1.79 | 4.16 | 4.20 | 2.51 | 2.33 | 5.91 | 4.59 | 4.24 | 3.55 | 5.94 | 4.26 | 2.92 | 2.28 | 1.80 | 0.96 | 1.67 | 1.07 |
| 1E0N | 37 | 6.85 | 6.42 | 3.73 | 3.75 | 6.79 | 6.29 | 5.10 | 5.19 | 7.88 | 3.12 | 4.45 | 1.99 | 8.51 | 3.27 | 6.64 | 2.15 | 9.61 | 3.17 | 9.15 | 3.25 |
| 1I6C | 39 | 4.52 | 2.03 | 3.68 | 1.92 | 6.98 | 4.32 | 3.57 | 1.75 | 6.20 | 4.48 | 4.91 | 2.75 | 5.36 | 3.57 | 3.55 | 2.35 | 3.67 | 1.81 | 4.55 | 1.89 |
| 1JRJ | 39 | 2.22 | 1.31 | 2.06 | 1.23 | 3.72 | 2.71 | 3.06 | 2.57 | 3.05 | 2.68 | 2.16 | 1.46 | 3.57 | 2.98 | 2.35 | 1.63 | 1.16 | 0.39 | 1.62 | 0.34 |
| 1WR3 | 36 | 1.47 | 1.26 | 1.47 | 1.26 | 5.01 | 3.39 | 2.61 | 1.80 | 5.29 | 4.16 | 3.50 | 2.71 | 3.58 | 2.81 | 2.59 | 1.77 | 1.31 | 0.74 | 1.64 | 0.90 |
| 1WR4 | 36 | 7.36 | 6.67 | 2.22 | 1.64 | 5.36 | 3.67 | 2.08 | 1.67 | 4.25 | 3.74 | 3.22 | 2.77 | 3.38 | 2.63 | 2.45 | 1.57 | 1.29 | 0.65 | 1.39 | 0.76 |
| 1WY3 | 35 | 1.39 | 1.44 | 1.35 | 1.32 | 4.26 | 3.58 | 3.18 | 2.60 | 4.99 | 4.99 | 3.83 | 3.98 | 3.10 | 3.10 | 2.30 | 2.30 | 0.61 | 0.61 | 0.68 | 0.68 |
| 1YIU | 37 | 2.11 | 1.86 | 2.00 | 1.61 | 5.67 | 4.91 | 2.90 | 2.40 | 6.09 | 5.19 | 3.26 | 2.92 | 4.39 | 4.33 | 3.15 | 2.69 | 1.31 | 0.79 | 1.20 | 0.98 |
| 1YYB | 27 | 2.65 | 1.57 | 1.89 | 1.03 | 3.26 | 1.29 | 1.85 | 1.00 | 2.39 | 1.24 | 1.57 | 0.69 | *no contacts predicted* | | | | 2.02 | 1.07 | 2.41 | 1.36 |
| 2BN6 | 33 | 2.17 | 1.01 | 1.62 | 0.95 | 12.44 | 9.69 | 8.32 | 6.42 | 3.53 | 1.68 | 2.38 | 1.38 | 2.53 | 1.39 | 1.75 | 1.06 | 2.01 | 1.01 | 3.18 | 1.41 |
| 2GDL | 31 | 6.32 | 4.90 | 4.94 | 4.07 | 7.36 | 4.69 | 5.01 | 4.14 | 5.80 | 3.63 | 3.99 | 2.36 | *no contacts predicted* | | | | 3.80 | 2.85 | 10.94 | 10.50 |
| 2K76 | 30 | 9.91 | 6.75 | 5.95 | 4.46 | 4.40 | 3.77 | 1.29 | 1.03 | 2.63 | 1.89 | 1.55 | 1.12 | 2.87 | 2.32 | 1.58 | 1.25 | 0.90 | 0.62 | 1.22 | 0.87 |
| 2KBL | 29 | 2.35 | 2.43 | 1.92 | 1.87 | 7.50 | 5.79 | 3.35 | 2.14 | 6.07 | 6.06 | 4.00 | 3.42 | 5.84 | 5.17 | 4.18 | 4.09 | 4.91 | 4.72 | 5.01 | 4.67 |
| 2KI0 | 36 | 11.92 | 10.82 | 8.38 | 8.48 | 2.51 | 1.89 | 2.18 | 1.80 | 2.62 | 2.31 | 1.87 | 1.52 | 1.74 | 1.29 | 1.31 | 0.99 | 2.01 | 1.40 | 3.44 | 2.60 |
| 2KYA | 34 | 3.37 | 1.25 | 3.09 | 0.97 | 8.52 | 6.61 | 6.74 | 4.35 | 4.71 | 1.89 | 3.72 | 0.98 | 6.40 | 2.96 | 3.51 | 1.11 | 5.11 | 4.61 | 6.08 | 5.33 |
| 2L0G | 32 | 3.73 | 1.82 | 2.27 | 1.36 | 3.57 | 1.48 | 1.70 | 1.04 | 3.62 | 2.23 | 2.55 | 1.21 | 2.92 | 1.85 | 2.03 | 1.13 | 1.61 | 0.81 | 2.72 | 1.03 |
| 2OVC | 33 | 2.89 | - | 2.40 | - | 10.39 | - | 7.16 | - | 8.13 | 8.31 | 1.48 | 0.82 | *no contacts predicted* | | | | 2.48 | 0.31 | 2.61 | 0.30 |
| 2YSC | 39 | 5.03 | 4.81 | 4.07 | 3.89 | 5.96 | 4.98 | 3.76 | 2.57 | 5.53 | 5.30 | 4.01 | 3.77 | 3.94 | 3.92 | 2.91 | 2.81 | 1.89 | 1.10 | 2.89 | 1.42 |
| 2YSF | 40 | 4.67 | 3.59 | 3.86 | 3.56 | 6.98 | 5.08 | 3.77 | 2.29 | 5.79 | 5.11 | 3.42 | 2.47 | 3.89 | 3.33 | 3.03 | 2.32 | 1.51 | 0.51 | 2.12 | 0.66 |
| 2YSG | 40 | 8.99 | 8.32 | 3.89 | 3.15 | 6.32 | 4.68 | 3.42 | 2.24 | 6.07 | 5.02 | 3.74 | 3.21 | 5.63 | 3.99 | 2.55 | 1.93 | 2.06 | 0.90 | 2.70 | 0.88 |
| 2YSH | 40 | 4.09 | 2.74 | 3.44 | 1.99 | 6.18 | 3.74 | 3.51 | 2.23 | 7.45 | 4.18 | 4.88 | 3.22 | 5.77 | 3.77 | 4.05 | 2.42 | 2.38 | 1.21 | 4.42 | 1.33 |
| 2YSI | 40 | 4.42 | 3.52 | 4.19 | 3.35 | 8.64 | 7.92 | 3.77 | 2.86 | 6.61 | 6.85 | 4.60 | 4.23 | 7.08 | 5.87 | 3.87 | 3.37 | 3.68 | 1.60 | 4.39 | 2.80 |
| AVG | | **4.49** | **3.29** | **3.19** | **2.34** | **5.94** | **4.24** | **3.63** | **2.49** | **5.44** | **3.96** | **3.51** | **2.37** | **4.81** | **3.41** | **3.05** | **2.00** | **2.80** | **1.63** | **3.59** | **2.16** |

**Table S10**. Dataset 3: long linear peptides

|  |  | **PEP-FOLD 3.5** | | | | **CABS-flex  *de novo*** | | | | **CABS-flex *contacts*** | | | | **Alpha Fold** | | **ESM Fold** | |
| --- | --- | --- | --- | --- | --- | --- | --- | --- | --- | --- | --- | --- | --- | --- | --- | --- | --- |
| PDB | LEN | PRIME | | BEST | | PRIME | | BEST | | PRIME | | BEST | | PRIME | | PRIME | |
| 1DV0 | 47 | 7.83 | 7.87 | 4.45 | 4.56 | 4.31 | 4.14 | 3.88 | 3.29 | 4.79 | 4.48 | 4.41 | 4.30 | 4.75 | 4.68 | 4.54 | 4.77 |
| 1F4I | 45 | 3.40 | 2.99 | 2.75 | 1.85 | 3.82 | 2.97 | 2.92 | 2.41 | 2.16 | 1.52 | 1.76 | 1.16 | 1.80 | 0.60 | 1.21 | 0.82 |
| 1IFY | 49 | 8.74 | 8.28 | 4.82 | 3.27 | 4.30 | 3.31 | 3.06 | 2.51 | 2.86 | 1.93 | 2.04 | 1.42 | 1.23 | 0.87 | 1.83 | 1.38 |
| 1K1V | 41 | 1.42 | 1.42 | 1.09 | 1.09 | 4.19 | 4.19 | 2.57 | 2.49 | 2.87 | 2.87 | 2.05 | 2.05 | 0.67 | 0.67 | 0.83 | 0.83 |
| 1ND9 | 49 | 4.57 | 4.60 | 4.24 | 4.23 | 6.12 | 6.15 | 4.90 | 4.92 | 4.48 | 4.53 | 3.88 | 3.91 | 2.85 | 2.88 | 3.90 | 3.92 |
| 1P9C | 45 | 5.35 | 1.88 | 5.09 | 1.78 | 7.26 | 2.92 | 5.13 | 0.89 | 7.94 | 2.44 | 5.27 | 1.61 | 5.13 | 1.63 | 6.59 | 1.96 |
| 1PGY | 47 | 3.32 | 3.04 | 2.82 | 2.22 | 4.89 | 3.92 | 3.78 | 3.21 | 5.21 | 4.83 | 3.05 | 2.80 | 2.62 | 1.60 | 3.02 | 2.61 |
| 1PV0 | 46 | 3.12 | 2.28 | 2.18 | 1.67 | 3.22 | 2.57 | 2.80 | 1.98 | 2.42 | 1.95 | 1.93 | 1.43 | 2.10 | 1.16 | 2.34 | 1.39 |
| 1USE | 47 | 2.15 | - | 1.16 | - | 5.30 | 3.95 | 2.71 | 2.10 | *no contacts predicted* | | | | 2.14 | 0.78 | 2.20 | 0.93 |
| 1VPU | 45 | 6.60 | 5.24 | 5.13 | 3.88 | 5.17 | 4.01 | 4.26 | 2.96 | 5.43 | 4.69 | 4.06 | 3.25 | 5.84 | 4.83 | 9.91 | 9.48 |
| 1W4E | 47 | 5.86 | 5.14 | 5.26 | 4.81 | 5.06 | 4.98 | 4.29 | 4.20 | 4.88 | 5.35 | 4.09 | 4.37 | 5.04 | 5.00 | 4.94 | 5.13 |
| 1W4G | 47 | 5.34 | 5.06 | 4.62 | 4.74 | 5.20 | 5.60 | 4.26 | 4.25 | 4.66 | 5.51 | 4.19 | 4.60 | 4.82 | 5.08 | 4.72 | 5.21 |
| 1W4H | 47 | 9.21 | 6.32 | 4.81 | 5.23 | 6.13 | 5.63 | 4.27 | 4.32 | 4.85 | 4.91 | 4.25 | 4.28 | 4.82 | 5.02 | 5.14 | 5.18 |
| 1WR7 | 41 | 3.71 | 2.65 | 2.86 | 1.40 | 5.51 | 4.75 | 3.73 | 2.88 | 4.97 | 3.47 | 2.95 | 2.02 | 1.86 | 0.64 | 1.86 | 0.71 |
| 1YMZ | 43 | 5.07 | 3.50 | 2.57 | 1.68 | 9.00 | 5.04 | 6.16 | 3.28 | 8.52 | 5.20 | 9.12 | 4.43 | 10.96 | 7.63 | 11.11 | 7.61 |
| 1YWJ | 41 | 3.81 | - | 2.09 | - | 5.60 | 4.19 | 3.37 | 1.82 | 4.12 | 3.12 | 3.68 | 2.26 | 5.66 | 4.68 | 7.52 | 6.44 |
| 2BTG | 47 | 5.30 | 5.13 | 4.72 | 5.10 | 5.05 | 5.07 | 4.39 | 4.26 | 4.97 | 4.94 | 4.30 | 4.33 | 4.87 | 5.09 | 4.99 | 5.19 |
| 2DMV | 43 | 5.32 | 3.04 | 3.75 | 1.73 | 6.67 | 4.01 | 4.87 | 3.27 | 5.29 | 3.22 | 3.97 | 2.19 | 2.82 | 0.89 | 5.58 | 1.03 |
| 2E5T | 46 | 1.59 | 1.35 | 1.39 | 0.99 | 3.93 | 3.59 | 2.93 | 2.91 | 1.76 | 1.60 | 1.51 | 1.40 | 1.08 | 1.05 | 1.89 | 1.86 |
| 2EKK | 47 | 2.80 | 1.23 | 2.11 | 1.23 | 3.18 | 2.50 | 2.91 | 2.33 | 2.48 | 1.30 | 1.33 | 0.88 | 1.55 | 0.48 | 2.01 | 0.77 |
| 2J8P | 49 | 8.67 | 8.96 | 3.95 | 3.67 | 5.52 | 4.94 | 3.53 | 3.28 | 5.04 | 4.50 | 4.21 | 3.87 | 7.11 | 7.04 | 10.26 | 10.48 |
| 2JNH | 46 | 6.38 | 5.75 | 5.55 | 4.82 | 4.51 | 4.79 | 3.34 | 3.76 | 4.73 | 4.42 | 4.34 | 4.00 | 4.53 | 4.65 | 4.56 | 4.64 |
| 2K9D | 44 | 3.94 | 1.51 | 2.99 | 1.51 | 4.33 | 2.85 | 3.83 | 2.08 | 3.37 | 3.11 | 3.04 | 2.90 | 3.73 | 7.44 | 3.70 | 7.56 |
| 2L4J | 46 | 6.52 | 2.23 | 4.94 | 1.97 | 7.23 | 2.93 | 5.61 | 2.45 | 7.16 | 3.19 | 3.59 | 2.07 | 3.39 | 1.15 | 4.01 | 1.37 |
| 2P81 | 44 | 4.37 | 1.83 | 4.37 | 1.83 | 6.18 | 3.96 | 3.96 | 2.48 | 4.06 | 2.27 | 3.30 | 1.67 | 3.05 | 2.04 | 6.51 | 2.62 |
| 2WXC | 47 | 3.16 | 2.74 | 3.16 | 2.47 | 4.09 | 3.68 | 3.49 | 3.07 | 2.38 | 1.75 | 1.81 | 1.44 | 1.52 | 0.78 | 1.97 | 1.00 |
| 2YSB | 49 | 7.04 | 4.51 | 4.72 | 2.74 | 6.52 | 5.50 | 5.46 | 3.88 | 4.90 | 3.32 | 3.51 | 2.04 | 2.08 | 0.54 | 3.92 | 0.51 |
| 2ZAJ | 49 | 9.38 | 4.38 | 8.12 | 4.15 | 8.34 | 5.41 | 5.85 | 4.10 | 7.07 | 5.21 | 4.78 | 3.33 | 2.42 | 1.08 | 5.99 | 1.65 |
| 3E21 | 45 | 2.98 | - | 2.98 | - | 4.67 | 2.65 | 3.79 | 2.11 | 4.33 | 1.33 | 4.18 | 1.07 | 4.49 | 0.50 | 4.46 | 1.12 |
| AVG | | **5.07** | **3.96** | **3.75** | **2.87** | **5.36** | **4.14** | **4.00** | **3.02** | **4.59** | **3.48** | **3.56** | **2.66** | **3.62** | **2.77** | **4.53** | **3.38** |

**Table S11**. Dataset 4: cyclic peptides closed by disulfide bridge

|  |  | **APPTEST** | | | | **PEP-FOLD 2** | | | | **CABS-flex *de novo*** | | | | **Alpha Fold** | | **ESM Fold** | |
| --- | --- | --- | --- | --- | --- | --- | --- | --- | --- | --- | --- | --- | --- | --- | --- | --- | --- |
| PDB | LEN | PRIME | | BEST | | PRIME | | BEST | | PRIME | | BEST | | PRIME | | PRIME | |
| 1B45 | 14 | 1.75 | 1.21 | 1.36 | 1.13 | 3.85 | 3.47 | 1.35 | 1.14 | 2.20 | 1.89 | 1.04 | 0.65 | 1.05 | 0.70 | 1.65 | 1.14 |
| 1ETL | 12 | 2.05 | - | 1.33 | - | 3.28 | - | 1.67 | - | 1.88 | - | 0.87 | - | 1.63 | - | 3.22 | - |
| 1GNB | 13 | 2.44 | 1.91 | 1.97 | 1.32 | 3.72 | 2.75 | 2.78 | 1.49 | 1.97 | 0.77 | 1.11 | 0.30 | 3.38 | 1.36 | 3.51 | 1.80 |
| 1HJE | 13 | 1.43 | - | 1.41 | - | 4.24 | - | 2.32 | - | 2.84 | - | 1.21 | - | 1.47 | - | 1.44 | - |
| 1HP9 | 22 | 1.91 | - | 1.90 | - | 2.97 | - | 2.14 | - | 2.34 | - | 1.22 | - | 1.94 | - | 2.06 | - |
| 1IEN | 19 | 2.35 | 1.87 | 1.56 | 1.45 | 4.99 | 4.71 | 2.30 | 2.04 | 2.86 | 2.76 | 1.36 | 1.31 | 1.76 | 1.10 | 1.38 | 0.56 |
| 1IM1 | 12 | 1.46 | - | 0.98 | - | 2.12 | - | 1.35 | - | 1.99 | - | 0.88 | - | 0.60 | - | 0.62 | - |
| 1IM7 | 13 | 3.15 | - | 2.08 | - | 4.34 | - | 2.63 | - | 2.16 | - | 1.64 | - | 1.67 | - | 8.51 | - |
| 1JBL | 14 | 1.00 | - | 1.00 | - | 1.86 | - | 1.86 | - | 2.57 | - | 1.60 | - | 0.37 | - | 0.71 | - |
| 1KCN | 21 | 2.72 | 2.46 | 2.41 | 2.08 | 6.39 | 6.64 | 2.69 | 2.44 | 2.10 | 1.78 | 2.05 | 1.39 | 3.08 | 2.88 | 4.16 | 4.12 |
| 1KWD | 16 | 2.56 | 2.30 | 1.93 | 1.57 | 2.54 | 2.03 | 1.53 | 1.24 | 2.55 | 2.12 | 1.32 | 1.25 | 1.98 | 1.74 | 1.82 | 1.62 |
| 1MII | 16 | 1.17 | - | 1.10 | - | 4.25 | - | 1.21 | - | 2.86 | - | 1.59 | - | 1.31 | - | 0.77 | - |
| 1MMC | 30 | 2.36 | 2.28 | 1.71 | 1.64 | 5.49 | 5.28 | 4.41 | 3.96 | 5.96 | 5.56 | 4.16 | 4.08 | 1.05 | 0.87 | 1.21 | 1.08 |
| 1N0A | 11 | 2.38 | - | 0.90 | - | 0.57 | - | 0.55 | - | 1.62 | - | 0.74 | - | 0.37 | - | 0.51 | - |
| 1N0C | 10 | 0.74 | - | 0.59 | - | 0.68 | - | 0.64 | - | 0.82 | - | 0.58 | - | 0.36 | - | 0.39 | - |
| 1NIM | 17 | 3.14 | 2.48 | 2.42 | 1.54 | 3.87 | 2.80 | 3.04 | 2.10 | 2.67 | 1.94 | 1.61 | 0.87 | 2.59 | 1.11 | 2.72 | 1.31 |
| 1OIG | 24 | 2.98 | 2.49 | 2.80 | 2.32 | 6.11 | 5.04 | 4.38 | 4.00 | 4.54 | 4.41 | 3.90 | 3.31 | 0.69 | 0.53 | 0.79 | 0.64 |
| 1ORX | 24 | 1.19 | - | 1.13 | - | 4.10 | - | 3.16 | - | 3.79 | - | 2.53 | - | 0.58 | - | 0.66 | - |
| 1R8T | 15 | 1.89 | 1.79 | 1.68 | 1.56 | 3.55 | 3.59 | 2.28 | 2.33 | 2.58 | 2.43 | 1.44 | 1.33 | 1.83 | 1.84 | 2.49 | 2.33 |
| 1RPC | 21 | 1.54 | 1.53 | 1.54 | 1.53 | 5.92 | 5.86 | 4.60 | 4.23 | 5.07 | 5.04 | 3.02 | 3.05 | 5.07 | 5.12 | 6.42 | 6.31 |
| 1SP7 | 24 | 3.20 | - | 2.61 | - | 6.87 | - | 2.56 | - | 3.38 | - | 2.13 | - | 1.31 | - | 1.62 | - |
| 1TER | 21 | 4.33 | 3.77 | 4.33 | 3.77 | 5.32 | 5.36 | 2.89 | 2.55 | 3.85 | 3.33 | 1.88 | 1.61 | 2.93 | 2.77 | 3.05 | 2.64 |
| 1V5A | 28 | 1.81 | 1.78 | 1.74 | 1.70 | 6.25 | 6.37 | 3.45 | 3.58 | 4.45 | 4.60 | 3.36 | 3.32 | 1.04 | 0.90 | 1.17 | 0.99 |
| 1V6R | 21 | 4.99 | - | 3.87 | - | 5.69 | - | 3.56 | - | 4.08 | - | 2.32 | - | 3.43 | - | 4.29 | - |
| 1WM8 | 28 | 2.30 | - | 2.21 | - | 6.67 | - | 4.43 | - | 4.36 | - | 2.81 | - | 2.76 | - | 2.69 | - |
| 1WQC | 26 | 3.08 | - | 2.25 | - | 2.12 | - | 1.43 | - | 2.46 | - | 1.51 | - | - | - | 2.33 | - |
| 1X7K | 19 | 3.63 | 1.97 | 2.17 | 1.52 | 4.95 | 3.31 | 4.37 | 2.49 | 1.96 | 0.97 | 1.82 | 0.42 | 1.92 | 0.45 | 1.93 | 0.52 |
| 1XGB | 13 | 2.70 | 2.67 | 1.75 | 1.68 | 3.02 | 3.02 | 2.56 | 2.56 | 2.50 | 2.22 | 1.55 | 1.21 | 2.80 | 2.72 | 2.85 | 2.93 |
| 2AJW | 22 | 1.79 | 0.64 | 1.55 | 0.60 | 3.01 | 1.67 | 1.75 | 0.72 | 3.35 | 3.49 | 2.08 | 2.15 | 2.25 | 2.00 | 1.21 | 1.22 |
| 2EFZ | 16 | 5.03 | - | 1.75 | - | 3.07 | - | 2.06 | - | 2.51 | - | 1.36 | - | 3.29 | - | 3.91 | - |
| 2I28 | 13 | 2.14 | - | 1.65 | - | 1.51 | - | 1.51 | - | 2.10 | - | 0.94 | - | 1.60 | - | 2.15 | - |
| 2IT7 | 28 | 1.67 | - | 1.46 | - | 4.02 | - | 3.13 | - | 4.32 | - | 3.04 | - | 0.36 | - | 0.37 | - |
| 2NX7 | 28 | 5.94 | 6.04 | 3.52 | 3.53 | 6.59 | 6.63 | 5.12 | 4.92 | 3.72 | 3.72 | 2.67 | 2.61 | 6.17 | 6.26 | 5.09 | 4.46 |
| 2OQ9 | 24 | 7.89 | 4.09 | 5.04 | 2.76 | 7.82 | 3.56 | 6.16 | 3.45 | 4.83 | 2.68 | 2.55 | 1.73 | - | - | 4.21 | 3.17 |
| AVG | | **2.67** | **2.43** | **1.99** | **1.86** | **4.17** | **4.24** | **2.70** | **2.66** | **3.04** | **2.92** | **1.88** | **1.80** | **1.96** | **2.02** | **2.41** | **2.17** |

**Table S12**. Dataset 5: cyclic peptides closed by backbone

|  |  | **APPTEST** | | | | **PEP-FOLD 2** | | | | **CABS-flex  *de novo*** | | | | **CABS-flex *contacts*** | | | | **Alpha Fold** | | **ESM Fold** | |
| --- | --- | --- | --- | --- | --- | --- | --- | --- | --- | --- | --- | --- | --- | --- | --- | --- | --- | --- | --- | --- | --- |
| PDB | LEN | PRIME | | BEST | | PRIME | | BEST | | PRIME | | BEST | | PRIME | | BEST | | PRIME | | PRIME | |
| 1HVZ | 18 | 0.88 | 0.67 | 0.88 | 0.58 | 3.73 | 3.16 | 2.45 | 1.74 | 2.43 | 1.95 | 1.90 | 1.12 | *no contacts predicted* | | | | 0.80 | 0.58 | 1.46 | 1.00 |
| 1IB9 | 34 | 1.95 | 1.54 | 1.92 | 1.54 | 6.08 | 5.05 | 4.40 | 3.62 | 5.02 | 4.77 | 3.58 | 3.11 | 4.44 | 3.81 | 2.93 | 2.71 | 3.46 | 0.84 | 3.69 | 1.02 |
| 1JJZ | 29 | 1.80 | 1.80 | 1.80 | 1.80 | 6.45 | 6.45 | 3.27 | 3.27 | 4.30 | 4.30 | 3.30 | 3.30 | 4.93 | 4.93 | 2.88 | 2.88 | 1.40 | 1.40 | 3.05 | 3.05 |
| 1N1U | 29 | 1.63 | 1.63 | 1.63 | 1.63 | 7.67 | 7.67 | 4.35 | 4.35 | 5.22 | 5.22 | 4.56 | 4.56 | 4.42 | 4.42 | 3.04 | 3.04 | 0.95 | 0.95 | 1.16 | 1.16 |
| 1NBJ | 30 | 1.49 | 1.49 | 1.49 | 1.49 | 3.99 | 3.99 | 3.99 | 3.99 | 4.33 | 4.33 | 2.88 | 2.88 | 4.26 | 4.26 | 2.40 | 2.40 | 1.73 | 1.73 | 6.33 | 6.33 |
| 1PXQ | 34 | 3.11 | 2.49 | 2.94 | 2.49 | 7.20 | 5.11 | 6.67 | 5.11 | 6.76 | 6.32 | 3.62 | 3.29 | *no contacts predicted* | | | | 5.66 | 6.09 | 12.52 | 7.09 |
| 1T9E | 14 | 3.68 | 3.68 | 3.48 | 3.48 | 4.48 | 4.48 | 2.88 | 2.88 | 2.66 | 2.66 | 1.46 | 1.46 | *no contacts predicted* | | | | 10.98 | 10.98 | 10.86 | 10.86 |
| 2F2I | 29 | 0.93 | 0.93 | 0.93 | 0.93 | 5.26 | 5.26 | 4.73 | 4.73 | 6.91 | 6.91 | 3.20 | 3.20 | 4.38 | 4.38 | 3.45 | 3.45 | 0.95 | 0.95 | 1.19 | 1.19 |
| 2KNM | 30 | 2.16 | 2.16 | 1.97 | 1.97 | 6.79 | 6.79 | 4.04 | 4.04 | 4.16 | 4.16 | 3.40 | 3.40 | 4.30 | 4.30 | 3.39 | 3.39 | 0.76 | 0.76 | 1.00 | 1.00 |
| 2KUX | 30 | 2.10 | 2.10 | 1.76 | 1.76 | 5.87 | 5.87 | 4.39 | 4.39 | 4.14 | 4.14 | 2.46 | 2.46 | 3.61 | 3.61 | 2.61 | 2.61 | 0.44 | 0.44 | 0.55 | 0.55 |
| 1BH4* | 30 | 1.71 | 1.54 | 1.43 | 1.37 | 5.96 | 6.22 | 4.39 | 4.52 | 4.42 | 4.41 | 3.33 | 3.33 | 3.36 | 3.23 | 2.70 | 2.58 | 1.56 | 1.43 | 1.62 | 1.56 |
| 1PT4* | 29 | 0.96 | 0.96 | 0.93 | 0.93 | 6.45 | 6.45 | 4.37 | 4.37 | 5.92 | 5.92 | 4.07 | 4.07 | 4.19 | 4.19 | 2.74 | 2.74 | 0.57 | 0.57 | 2.25 | 2.25 |
| 1R1F* | 37 | 1.81 | 1.78 | 1.81 | 1.78 | 4.53 | 4.46 | 4.53 | 4.46 | 5.69 | 5.70 | 3.28 | 3.25 | 4.13 | 4.05 | 3.02 | 3.03 | 2.63 | 2.33 | 4.70 | 4.56 |
| 1VB8* | 30 | 1.32 | 1.34 | 1.32 | 1.34 | 6.23 | 6.12 | 4.06 | 4.09 | 5.41 | 5.43 | 3.42 | 3.43 | 4.51 | 4.53 | 2.54 | 2.55 | 1.65 | 1.67 | 6.13 | 6.17 |
| 1YP8* | 33 | 1.73 | 1.73 | 1.41 | 1.41 | 6.67 | 6.67 | 4.82 | 4.82 | 6.60 | 6.60 | 3.80 | 3.80 | 4.25 | 4.25 | 3.45 | 3.45 | 1.20 | 1.20 | 3.05 | 3.05 |
| 1ZA8* | 31 | 1.54 | 1.54 | 1.40 | 1.40 | 6.18 | 6.18 | 4.41 | 4.41 | 5.20 | 5.20 | 3.82 | 3.82 | 3.74 | 3.74 | 2.62 | 2.62 | 1.86 | 1.86 | 9.98 | 9.98 |
| 2B38* | 31 | 1.33 | 1.07 | 1.30 | 1.05 | 5.28 | 4.69 | 3.47 | 3.24 | 4.69 | 4.57 | 3.34 | 3.01 | 3.76 | 3.56 | 2.70 | 2.29 | 0.91 | 0.89 | 3.05 | 1.00 |
| 2BEY* | 16 | 1.97 | 1.97 | 1.88 | 1.88 | 4.41 | 4.41 | 3.46 | 3.46 | 2.70 | 2.70 | 1.94 | 1.94 | *no contacts predicted* | | | | 4.94 | 4.94 | 9.19 | 9.19 |
| 2ERI* | 31 | 1.69 | 1.69 | 1.47 | 1.47 | 5.15 | 5.15 | 3.96 | 3.96 | 4.51 | 4.51 | 3.40 | 3.40 | 4.11 | 4.11 | 2.83 | 2.83 | 1.21 | 1.21 | 1.73 | 1.73 |
| 2F2J* | 29 | 0.76 | 0.76 | 0.76 | 0.76 | 5.70 | 5.70 | 4.80 | 4.80 | 5.65 | 5.65 | 3.49 | 3.49 | 3.98 | 3.98 | 3.42 | 3.42 | 0.70 | 0.70 | 1.06 | 1.06 |
| 2GJ0* | 31 | 1.40 | 1.40 | 1.24 | 1.24 | 4.34 | 4.41 | 4.23 | 4.24 | 3.86 | 3.83 | 3.22 | 3.11 | 3.58 | 3.58 | 2.57 | 2.37 | 1.65 | 1.70 | 2.50 | 2.57 |
| 2K7G* | 29 | 0.83 | 0.83 | 0.83 | 0.83 | 8.14 | 8.14 | 3.63 | 3.63 | 4.06 | 4.06 | 3.19 | 3.19 | 3.64 | 3.64 | 2.30 | 2.30 | 2.03 | 2.03 | 0.67 | 0.67 |
| 2KUK* | 30 | 1.05 | 1.05 | 1.05 | 1.05 | 4.21 | 4.21 | 4.09 | 4.09 | 4.94 | 4.94 | 3.89 | 3.89 | 3.99 | 3.99 | 3.33 | 3.33 | 0.60 | 0.60 | 0.86 | 0.86 |
| 2KVX* | 28 | 1.54 | 1.54 | 1.45 | 1.45 | 4.23 | 4.23 | 4.10 | 4.10 | 4.55 | 4.55 | 2.87 | 2.87 | 3.79 | 3.79 | 2.03 | 2.03 | 0.73 | 0.73 | 0.94 | 0.94 |
| 2LAM* | 29 | 0.82 | 0.82 | 0.82 | 0.82 | 6.85 | 6.85 | 5.06 | 5.06 | 5.72 | 5.72 | 3.52 | 3.52 | 3.47 | 3.47 | 3.26 | 3.26 | 2.13 | 2.13 | 2.11 | 2.11 |
| 2LUR* | 29 | 1.41 | 1.41 | 1.41 | 1.41 | 5.63 | 5.63 | 3.65 | 3.65 | 5.11 | 5.11 | 3.42 | 3.42 | 4.84 | 4.84 | 3.12 | 3.12 | 2.08 | 2.08 | 4.45 | 4.45 |
| 2M9O* | 29 | 0.94 | 0.94 | 0.83 | 0.83 | 6.79 | 6.79 | 4.66 | 4.66 | 4.63 | 4.63 | 3.30 | 3.30 | 3.91 | 3.91 | 2.60 | 2.60 | 2.12 | 2.12 | 1.50 | 1.50 |
| 5WOW* | 39 | 1.55 | 1.30 | 1.55 | 1.30 | 7.43 | 7.21 | 4.37 | 3.23 | 5.78 | 4.31 | 3.87 | 3.28 | 5.48 | 4.58 | 4.21 | 3.69 | 4.27 | 2.19 | 4.02 | 2.17 |
| AVG | | **1.57** | **1.51** | **1.49** | **1.43** | **5.78** | **5.62** | **4.19** | **4.03** | **4.83** | **4.74** | **3.27** | **3.18** | **4.06** | **3.96** | **2.82** | **2.73** | **2.14** | **1.97** | **3.63** | **3.18** |

*These peptides were used in the APPTEST dataset for training the neural network.

**Table S13**. Dataset 6: cyclic peptides closed by backbone, cases from AfCycDesign (doi: 10.1101/2023.02.25.529956) and HighFold (doi: 10.1101/2023.08.27.554979) papers. The table contains a comparison of prime models between AfCycDesign, HighFold, MODPEP 2.0, and other methods tested in the manuscript. The AfCycDesign and HigFold methods used a different set of peptides for testing, so we only compared the methods on the common examples in the two benchmarks. In some cases, these two methods compared structure predictions to different reference structures from PDB (indicated by column PDB_alt). Given RMSD numbers are calculated on full structures (prime models). For MODPEP 2.0, the prime model was the best accuracy model out of the 10 predicted models, for other methods 10 or less, as described in the manuscript.

| PDB | PDB_alt | **APPTEST** | **PEPFOLD2** | **CABS-flex de novo** | **CABS-flex + RX** | **ESMFold** | **AlphaFold** | **AfCyc Design** | **HighFold** | **MODPEP2.0** |
| --- | --- | --- | --- | --- | --- | --- | --- | --- | --- | --- |
| 1HVZ | - | 0.88 | 3.73 | 2.43 | 2.43 | 1.46 | 0.80 | 1.23 | 2.32 | 1.03 |
| 1IB9 | 2PO8 | 1.95 | 6.08 | 5.02 | 4.44 | 3.69 | 3.46 | 1.96 | 3.63 | 8.04 |
| 1JJZ | 1ZNU | 1.80 | 6.45 | 4.30 | 4.93 | 3.05 | 1.40 | 0.57 | 0.70 | 7.69 |
| 1NBJ | 1DF6 | 1.49 | 3.99 | 4.33 | 4.26 | 6.33 | 1.73 | 2.13 | 1.04 | 7.05 |
| 2KNM | - | 2.16 | 6.79 | 4.16 | 4.30 | 1.00 | 0.76 | 0.89 | 1.02 | 7.34 |
| 2KUX | - | 2.10 | 5.87 | 4.14 | 3.61 | 0.55 | 0.44 | 0.52 | 0.90 | 4.88 |
| 1BH4 | - | 1.71 | 5.96 | 4.42 | 3.36 | 1.62 | 1.56 | 1.40 | 1.85 | 7.86 |
| 1PT4 | 2KCH | 0.96 | 6.45 | 5.92 | 4.19 | 2.25 | 0.57 | 0.50 | 0.66 | 6.88 |
| 1R1F | - | 1.81 | 4.53 | 5.69 | 4.13 | 4.70 | 2.63 | 2.44 | 2.46 | 8.63 |
| 1VB8 | - | 1.32 | 6.23 | 5.41 | 4.51 | 6.13 | 1.65 | 0.69 | 0.77 | 7.59 |
| 1ZA8 | - | 1.54 | 6.18 | 5.20 | 3.74 | 9.98 | 1.86 | 0.41 | 0.63 | 8.82 |
| 2B38 | - | 1.33 | 5.28 | 4.69 | 3.76 | 3.05 | 0.91 | 0.91 | 1.07 | 6.59 |
| 2ERI | - | 1.69 | 5.15 | 4.51 | 4.11 | 1.73 | 1.21 | 0.67 | 0.74 | 9.57 |
| 2GJ0 | - | 1.40 | 4.34 | 3.86 | 3.58 | 2.50 | 1.65 | 0.79 | 1.15 | 8.30 |
| 2K7G | - | 0.83 | 8.14 | 4.06 | 3.64 | 0.67 | 2.03 | 0.49 | 0.66 | 8,04 |
| 2KUK | - | 1.05 | 4.21 | 4.94 | 3.99 | 0.86 | 0.60 | 0.72 | 0.79 | 6.78 |
| 2KVX | - | 1.54 | 4.23 | 4.55 | 3.79 | 0.94 | 0.73 | 0.78 | 0.95 | 7.31 |
| 2LAM | - | 0.82 | 6.85 | 5.72 | 3.47 | 2.11 | 2.13 | 0.46 | 0.49 | 8.72 |
| 2LUR | - | 1.41 | 5.63 | 5.11 | 4.84 | 4.45 | 2.08 | 1.99 | 2.01 | 7.96 |
| 2M9O | - | 0.94 | 6.79 | 4.63 | 3.91 | 1.50 | 2.12 | 0.58 | 0.72 | 8.83 |
| 5WOW | - | 1.55 | 7.43 | 5.78 | 5.48 | 4.02 | 4.27 | 3.45 | 4.41 | 7.03 |
| AVG | | **1.44** | **5.73** | **4.71** | **4.02** | **2.98** | **1.65** | **1.12** | **1.38** | **7.34** |
